# Evolution of the miR-290–295/miR-371–373 Cluster Family Seed Repertoire

**DOI:** 10.1371/journal.pone.0108519

**Published:** 2014-09-30

**Authors:** Shuang Wu, Munevver Aksoy, Jianting Shi, Hristo Botev Houbaviy

**Affiliations:** Department of Cell Biology, Rowan University School of Osteopathic Medicine, Two Medical Center Drive, Stratford, NJ, United States of America; IPMC, CNRS UMR 7275 UNS, France

## Abstract

Expression of the mouse miR-290–295 cluster and its miR-371–373 homolog in human is restricted to early embryos, primordial germ cells, the germ line stem cell compartment of the adult testis and to stem cell lines derived from the early embryonic lineages. Sequencing data suggest considerable seed diversification between the seven homologous pre-miRNAs of miR-290–295 but it is not clear if all of the implied miR-290–295 seeds are also conserved in the human miR-371–373 cluster, which consists of only three homologous pre-miRNAs. By employing miRNA target reporters we show that most, if not all, seeds in miR-290–295 are represented in miR-371–373. In the mouse, pre-miR-290, pre-miR-292 and pre-miR-293 express subsets of the miRNA isoforms processed from the single human pre-miR-371. Comparison of the possible miR-290–295/miR-371–373 seed repertoires in placental mammals suggests a model for the evolution of this miRNA cluster family, which would be otherwise difficult to deduce based solely on pre-miRNA sequence comparisons. The conservation of co-expressed seeds that is characteristic of miR-290–295/miR-371–373 should be taken into account in models of the corresponding miRNA-target interaction networks.

## Introduction

Pre-miRNA duplication and the acquisition of new target specificities by the corresponding mature microRNAs are major driving forces behind microRNA evolution [1]–[3]. Such duplications often result in the formation of clusters consisting of homologous pre-miRNAs, which are co-transcribed into common primary transcripts (pri-miRNAs) [4]–[7]. The acquisition of novel targets by homologous miRNAs is due to sequence variation at positions 2–7 or 2–8 at their 5′-ends, known respectively as the 6 mer and 7 mer seed [8], [9]. Subtle differences in the secondary structures of the homologous pre-miRNA hairpins often result in shifts of the positions where the nucleases Drosha and Dicer cleave the hairpin stems. These alternative cleavage sites change the seeds directly by shifting the 5′-ends of the mature miRNAs or indirectly by causing different strands of the processed pre-miRNA stems to be loaded into the microRNA induced silencing complex (miRISC) according to the so-called Zamore rules [10]. miRNA isoforms with such alternative seeds have been shown to repress distinct sets of targets [6], [11]. Seed diversification can occur even within individual pre-miRNAs, which can simultaneously produce multiple active miRNAs. Morin et al. have coined the term isomiR to refer to such overlapping miRNA species with alternative 5′- and 3′- ends [12].

The miR-290–295 cluster in the mouse and its miR-371–373 homolog in human are the founding members of the miR-290–295/miR-371–373 cluster family [13]–[15]. Within miR-290–295 and miR-371–373 the individual pre-miRNA hairpin sequences are homologous to each other and this homology together with the conservation of the putative promoter element as well as the synteny of the corresponding genomic loci is used to define miR-290–295/miR-371–373 cluster homologs in other species [15].

miR-290–295 are the most abundant miRNAs in mouse embryonic stem (ES) cells where they comprise approximately a third of the total miRNA pool but their expression ceases rapidly upon retinoic acid induced differentiation *in vitro* [13], [16]. miR-290–295 are also expressed in trophoblastic stem (TS) cells and extraembryonic endoderm (XEN) cells [15], [17]. miR-371–373 are expressed in human ES cells albeit at low levels (1% or less of the miRNA pool) [12], [14]. miR-290–295 is among the first genes expressed after fertilization with *de novo* synthesis of the corresponding mature miRNAs commencing at the two cell stage [18]. As development proceeds miR-290–295/miR-371–373 expression becomes restricted to the germ line with high levels reported in primordial germ cells and the stem cell compartment of the adult testis [19]–[21]. The expression pattern of miR-290–295/miR-371–373 is, thus, consistent with functions during early embryonic development and/or the development of the germ line as well as the maintenance and/or differentiation of stem cell lines derived from the early embryonic lineages. Indeed, miR-290–295/miR-371–373 family members directly control the G1-S cell cycle transition and inhibit apoptosis due to genotoxic stress in mouse ES cells, increase the efficiency of both mouse and human somatic cell reprogramming to induced pluripotent (iPS) cells and indirectly control the methylation of the mouse ES cell genome [22]–[26]. Deletion of the miR-290–295 cluster results in partially penetrant embryonic lethality and female-specific sterility due to inefficient colonization of the embryonic gonad by primordial germ cells [20].

Sequencing data suggest that both the production of isomiRs with alternative 5′-ends and the loading of alternative strands of the pre-miRNA stems into miRISC cause considerable seed diversification of the homologous pre-miR-290–295 and pre-miR-371–373 hairpins [12], [16], [27]. However, discrepancies between the various datasets make it difficult to determine the strands of the pre-miRNA stems that produce active miRNAs and functional data are required to prove that any isomiRs with shifted 5′-ends implied by the sequencing data are indeed active. Furthermore it is not *a priori* clear whether all seeds that are implied by the sequencing data for the seven pre-miRNAs in the mouse miR-290–295 are also present in the three pre-miRNAs of the human miR-371–373. Here, we address these questions by studying the silencing of synthetic reporters targeted specifically by the various predicted miR-290–295 and miR-371–373 isoforms.

## Results

### The miR-290–295/miR-371–373 loci in placental mammals

**Figure 1:**
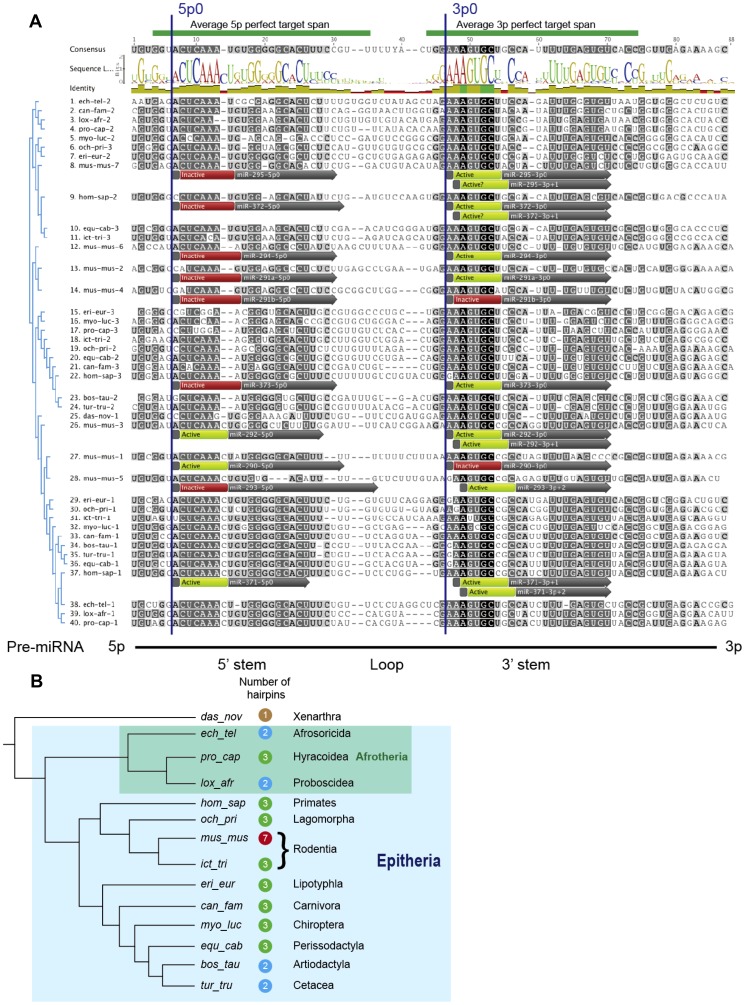
Comparisons of the miR-290–295/miR-371–373 clusters in *Placentalia*. (**A**) Multiple sequence alignment of the individual pre-miRNAs from species belonging to 14 distinct placental orders. Sequences are ordered according to the UPMGA tree (shown on the left) and are labeled with the species abbreviation. pre-miRNAs are numbered according to their position with respect to the transcription start site (the most promoter proximal pre-miRNAs are at position 1). The alignment consensus and sequence logo are shown at the top and the pre-miRNA secondary structure elements at the bottom. The 5p0 and 3p0 reference positions discussed in the text are also indicated. Active (light) and inactive (dark) miRNA seed positions within the human and mouse clusters are highlighted. The activities of miR-295-3p+1 and miR-372-3p+1 are unknown (Active?). Species abbreviations are as follows: bos-tau – *Bos taurus* (domestic cow), can-fam – *Canis familiaris* (dog), das-nov – *Dasypus novemcinctus* (armadillo), ech-tel – *Echinops telfairi* (lesser hedgehog), equ-cab – *Equus caballus* (horse), eri-eur – *Erinaceus europaeus* (European hedgehog), hom-sap – *Homo sapiens* (human), lox-afr – *Loxodonta africana* (African bush elephant), mus-mus – *Mus musculus* (house mouse), myo-luc – *Myotis lucifidus* (little brown bat), och-pri – *Ochotona princeps* (American pika), pro-cap – *Procavia capensis* (rock hyrax), ict-tri – *Ictidomys tridecemlineatus* (thirteen-lined ground squirrel), tur-tru – *Tursiops truncatus* (bottlenose dolphin) (**B**) Evolutionary relationships between the species in (A). Species abbreviations are followed by the number of pre-miRNA hairpins in the corresponding cluster. The names of orders and relevant superclades are indicated. The evolutionary tree is according to ref [29].

Previously miR-290–295/miR-371–373 homologs were identified in four distinct mammalian orders by a combination of BLAST and HMMER searches [15], [28]. Applying this strategy to the presently available genomic data identifies clustered pre-miRNAs that fit well the pre-miR-290-295/pre-miR-371–373 consensus in all sequenced Epitherian genomes as well as a single weakly similar pre-miRNA in the armadillo (Figure 1, armadillo  =  das-nov, sequence #25). However, as reported previously, neither BLAST nor HMMER identify miR-290–295/miR-371–373 homologs in the genomes of marsupials and non-mammalian vertebrates [15].

Extant placental mammals form clade *Placentalia* (its superclade *Eutheria* includes additional extinct species) which is split into two subclades, the South America-originating *Xenarthra* and *Epitheria* (containing most extant placental species) [29]. The discovery of a putative miR-290–295/miR-371–373 homolog in the armadillo which represents Xenarthrans and the absence of miR-290–295/miR-371–373 homologs in marsupials, thus, suggests that the miR-290–295/miR-371–373 cluster family has appeared after the marsupial-placental split of ancestral mammals (*Theria*) but possibly prior to the basal *Xenarthra*-*Epitheria* split of placentals.

While the seven-hairpin arrangement of the mouse miR-290–295 cluster is conserved in the rat genome (data not shown), in most species the miR-290–295/miR-371–373 clusters contain either three (the most common arrangement) or two (in orders *Artiodactyla*, *Afrosoricida* and *Proboscidea*) pre-miRNA hairpins (Figure 1B). In fact, the seven-hairpin structure of miR-290–295 is not even common to all rodents as evidenced by the three-hairpin organization of the locus in the squirrel (Figure 1B, ict_tri).

### miRNA isoform and seed nomenclature for the homologous pre-miR-290–295/pre-miR-371–373

To simplify further discussion we modify the standard 5p- and 3p- notation used to designate miRNA species processed from the two strands of the pre-miRNA stem to account for variations in their 5′- ends and we introduce a notation for the corresponding alternative seeds [7]. The proposed nomenclature is based on the miR-290–295/miR-371–373 multiple sequence alignment and can be applied to any cluster of homologous pre-miRNAs that yields alternative miRNA isoforms.

We designate the first nucleotide position within the conserved ACUCAAA block found in the 5′- strands of the pre-miR290–295/pre-miR-371–373 stems 5p0 and the first position of the conserved AAAGUGC block present in the 3′- strands of the pre-miRNAs 3p0 (Figure 1A). miRNAs which have 5′-ends shifted by N nucleotides to the right (towards the 3′-end of the pre-miRNAs) from the reference 5p0 and 3p0 positions are designated 5p+N and 3p+N respectively. Shifts to the left (i.e. towards the 5′-end of the pre-miRNA) are designated by negative numbers (5p-N and 3p-N).

The 6 mer seed sequences (positions 2–7) of any putative 5p0 and 3p0 miRNAs are invariant (they correspond to the highly conserved sequence blocks in Figure 1A and, as discussed below, any deviations from the consensus are not represented in active mature miRNAs). These 6 mer seeds are designated (5p)2–7 ( = CUCAAA) and (3p)2–7 ( = AAGUGC) respectively. The 7 mer (positions 2–8) seeds are given by listing the position 8 base following the 6 mer seed designation. The seeds of the 3p-N, 3p+N, 5p-N and 5p+N isoforms are given as positions within the conserved sequence blocks that remain in the miRNA seed followed or preceded by the bases outside of the conserved blocks that complete the seed (i.e. (3p)3-7CG, (3p)4-7CGC etc.).

### Available RNA sequencing data imply functional non-equivalence of the individual miR-290–295/miR-371–373 pre-miRNAs but are not sufficient to determine their precise seed repertoire

miR-290–295/miR-371–373 short RNA sequencing data include datasets representing total short RNA from mouse and human ES cells, total short RNA from ectopic overexpression experiments in HEK-293 cells as well as high-throughput sequencing of RNAs isolated by crosslinking immunoprecipitation (HITS-CLIP) data representing RNAs crosslinked to the Argonaute component of miRISC [6], [12], [16], [27].

**Figure 2:**
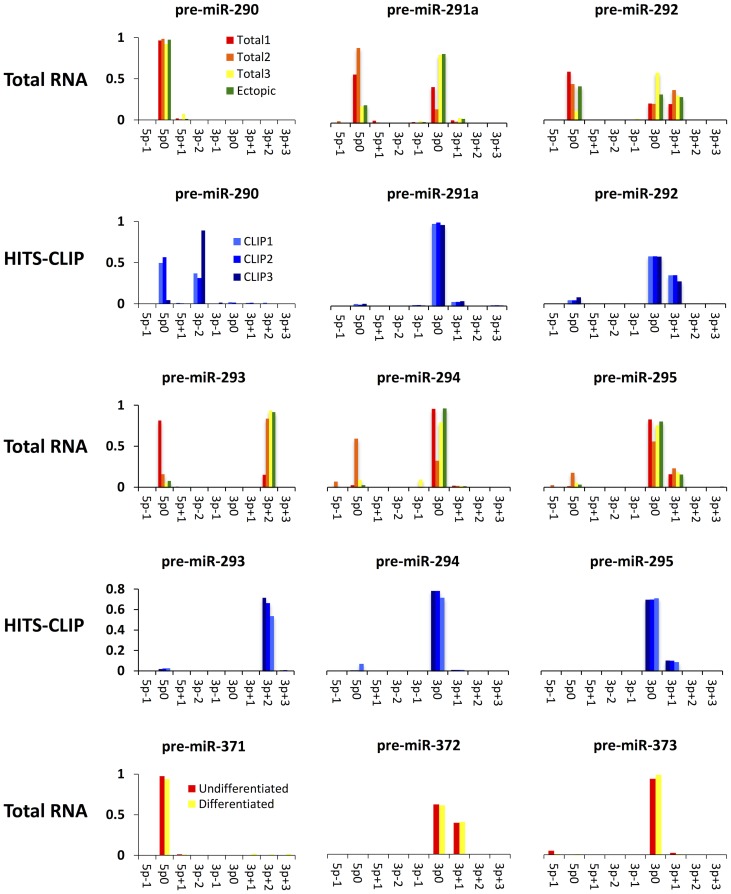
Short RNA 5′-end distributions in pre-miR-290–295 and pre-miR-371–373 sequencing data. The frequencies of observed 5′-ends of RNA reads in various short RNA sequencing datasets are plotted as a function of pre-miRNA sequence position. pre-miRNA sequence co-ordinates are given with respect to the 5p0 and 3p0 positions in Figure 1A and the sum of 5′-end frequencies is normalized to 1 for each individual pre-miRNA. For pre-miR-290-295 the top panels show total ES cell RNA and HEK-293 ectopic overexpression RNA sequencing data (Total1-3, Ectopic) and the bottom panels show HITS-CLIP data. Dataset Total1 is the total RNA dataset from ref. [27], Total 2 and Total 3 are respectively the J1 and Dcr^+/+^ total RNA datasets from ref. [16] and Total 4 is from ref. [6]. CLIP1-3 correspond to datasets WT1A, WT1B and WT2 from ref. [27]. The panels corresponding to pre-miR-371-373 show total RNA sequencing data from undifferentiated human ES cells (Undifferentiated) and human ES cells that have been differentiated into embryoid bodies (Differentiated) according to ref [12]. The data for pre-miR-291b, which yields very few reads in all datasets and is, thus, noisy is given in Figure S1.

Overall the 5′-ends of RNAs in the sequencing data map predominantly to the 5p0 and 3p0 positions of pre-miR-290–295/pre-miR-371–373 and more reads map to the 3p- than the 5p- strands of the hairpin stems. However, analysis of the data for the individual pre-miRNAs suggests considerable seed diversification both via alternative loading of 5p- and 3p- miRNA isoforms and via the production of isomiRs with alternative 5′- ends. (Figure 2, Figure S1). Reads that originate from the mouse pre-miR-290 and the human pre-miR-371 (the most upstream hairpins in the clusters) map predominantly to the 5′- strand of the hairpin stem, suggesting that miR-290-5p and miR-371-5p and not the corresponding 3p- isomiRs are the active mature miRNA species and most sequencing data imply that miR-293-3p+2 is the sole isomiR processed from pre-miR-293 (Figure 2, pre-miR-293). In addition, several hairpins yield two miRNA isoforms with alternative 5′-ends represented by similar numbers of reads in the sequencing libraries (Figure 2, pre-miR-292, pre-miR-295, pre-miR-372). However, discrepancies between the various datasets, make the unambiguous assignment of active mature miRNAs to each pre-miRNA hairpin difficult (Figure 2, total RNA datasets for pre-miR-291a, pre-miR-293, pre-miR-294 and total RNA versus HITS-CLIP data for pre-miR-290).

Together the 5p- and 3p- miRNAs processed from the mouse miR-290–295 cluster are capable of producing a total of between 5 and 9 distinct seeds depending on the seed definition, sequencing dataset and criteria used to identify active miRNA species (Compare Figure1A and Figure 2). The fact that in most species the miR-290–295/miR-371–373 clusters consist of only two or three pre-miRNA hairpins (Figure 1B), raises the question of whether all the distinct miRNA seeds that could potentially be encoded by the mouse miR-290–295 cluster are also encoded by its homologs in other species. The first step in addressing this question is the experimental identification of functionally active miRNAs within miR-290–295 and miR-371–373.

### Functional assignment of active miRNAs narrows down the potential seeds within the miR-290–295 cluster

The multiple sequence alignment of the miR-290–295/miR-371–373 pre-miRNAs reveals considerable variation in the middle of the predicted mature miRNA sequences (Figure 1, Figure S2). When the middle of the miRNA does not pair with the target mRNA, efficient silencing requires the presence of multiple miRNA binding sites [30], [31]. Thus, single perfectly complementary miR-290–295/miR-371–373 target sites should confer silencing that is specific to the individual pre-miRNA hairpins and can be used to determine the strand(s) of each hairpin stem that yield active miRNA species.

We implemented this strategy by inserting sequences perfectly complementary to miR-290–295 downstream of a firefly luciferase reporter driven by the CAG promoter [32]. The target sites correspond to defined 5′ and 3′ blocks within the pre-miR290–295/pre-miR-371–373 multiple sequence alignment and span all 5′- and 3′- isomiRs implied by the sequencing data (Figure 1A, Figure S2, Table S1).

**Figure 3:**
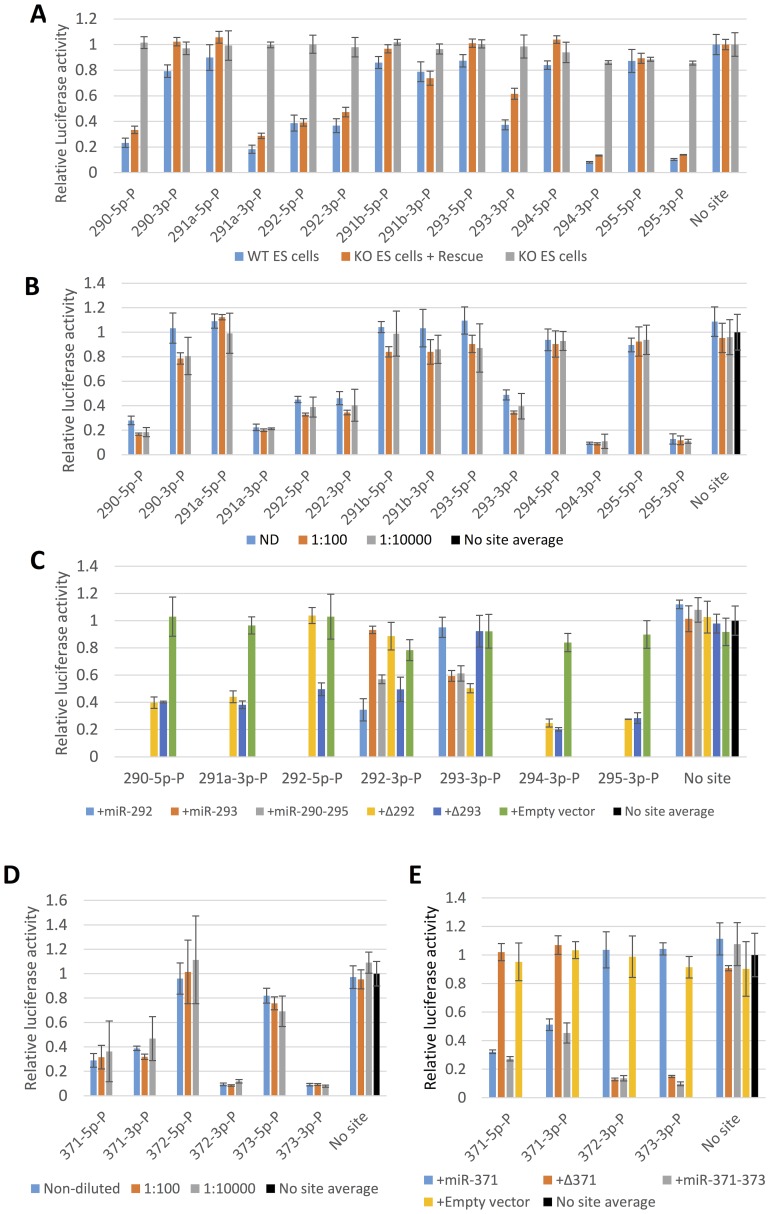
Silencing of luciferase reporters containing perfectly complementary miR-290–295 and miR-371–373 target sites in mouse ES cells. Reporter activities are expressed as firefly/*Renilla* luciferase activity ratios normalized to the control transfection of a firefly luciferase construct that does not contain miRNA target sites (No site). In panels that combine multiple experiments with separate no site controls, normalization is to the average of these controls (No site average). (**A**) Silencing of reporters containing sites perfectly complementary to the putative mature miR-290–295 in wild type mouse ES cells (WT ES Cells), in miR-290–295 knockout ES cells (KO ES cells) and miR-290–295 knockout ES cells co-transfected with a miR-290–295 expression construct (KO ES cells + Rescue). The target site sequences are summarized in Table 1. (**B**) The mixtures of firefly and *Renilla* luciferase constructs shown in A were serially diluted (ND  =  non-diluted, 1∶100, 1∶10000) with a plasmid expressing EGFP so that the total DNA concentration remains constant (to maintain the same transfection efficiency) and were transfected into wild type mouse ES cells. The results obtained from further dilution of the reporters are shown in Figure S3. (**C**) Reporter activities in miR-290–295 knockout ES cells co-transfected with the following rescue constructs: full-length miR-290–295 (+miR-290–295), pre-miR-292 deletion mutant (+Δ292), pre-miR-293 deletion mutant (+Δ293), single pre-miR-292 expression construct (+miR-292), single pre-miR-293 expression construct (+miR-293) and control expression vector backbone that does not express any miRNAs (+Empty vector). (**D**) Serial dilutions (ND  =  non-diluted, 1∶100, 1∶10000) of reporters containing target sites perfectly complementary to miR-371–373 were performed as in Figure 3C and were co-transfected with a miR-371–373 expression construct into miR-290–295 null mouse ES cells. (**E**) The miR-371–373 luciferase reporters were co-transfected with the following expression constructs: full-length miR-371–373 expression construct (+miR-371–373), pre-miR-371 deletion mutant (+Δ371), single pre-miR-371 expression construct (+miR-371) or an empty expression vector control (+Empty vector).

As expected, none of the reporters were significantly silenced when transfected in miR-290–295 knockout ES cells (Figure 3A, KO ES cells) [20], [26]. Transfection of the reporters into wild type ES cells resulted in dramatically different levels of silencing for the different targets (Figure 3A, WT ES cells). Decreasing the concentration of the transfected reporters by 5–6 orders of magnitude had no effect on silencing relative to a diluted no target control (Figure 3B, Figure S3A). This behavior rules out any dependence of silencing on the miRNA-target stoichiometry and is consistent with mathematical models, which predict that a fixed fraction of miRNA targets undergoing Ago2 catalyzed cleavage should escape silencing regardless of their expression levels [33].

From the 5p-reporters, only the miR-290-5p and miR-292-5p target sites confer robust silencing despite the fact that all 5p-reporters are highly similar to each other (Figure 1A, Figure S2). All of the 3p-reporters except miR-290-3p and miR-291b-3p were robustly silenced in wild type ES cells. Once again highly similar sequences confer qualitatively different levels of silencing (Figure 1A, Figure S2). Thus, the perfectly complementary miR-290-295 reporters are specific for their cognate pre-miRNAs.

Co-transfection of a miR-290–295 expression vector and the miR-290–295 luciferase reporters into miR-290–295 knockout ES cells quantitatively rescues silencing (Figure 3A, compare datasets “WT ES cells” and “KO ES cells + Rescue”). Thus, we were able to confirm that the perfectly complementary targets are silenced by their cognate miRNAs by performing rescues with various mutant miR-290–295 expression constructs (Figure 3C).

The discrepancies between the luciferase data presented above and the total RNA sequencing datasets are likely due to PCR amplification artifacts, whereas discrepancies with the HITS-CLIP data are best explained by a propensity of the 5p- miR-290–295 isoforms to crosslink much less efficiently to the Argonaute miRISC component than the 3p- miRNAs. The presence of many more 3p- sequences than 5p-sequences in the pre-miR-292 HITS-CLIP data is likely due to the inefficient crosslinking of the active miR-292-5p, whereas the crosslinking of the active miR-290-5p is probably so inefficient that it is close to the background library contamination by inactive miR-290-3p sequences resulting in similar abundance of pre-miR-290 5p- and 3p- reads in the HITS-CLIP dataset (Figure 2).

In summary, the reporter silencing data presented above unambiguously identify the strands of the pre-miR-290–295 stems that produce active miRNAs. From the 5p- short RNA species only miR-290-5p and miR-292-5p represent active miRNAs. These miRNAs share the same (5p)2-7C 7mer seed. Our functional validation eliminates miR-290-3p as an active miRNA, but the maximum theoretical 3p- seeds remain between 4 and 5, depending on seed definition. Finally, we note that neither the miR-291b-5p nor the miR-291b-3p reporters were silenced in any of the above experiments, which is consistent with the fact that pre-miR-291b sequences represent about 0.1% of all reads that map to the entire miR-290–295 locus in the various sequencing datasets (Figure S1, note that the 5′- end distributions are different in the different libraries and, thus, indicative of noise due to non-specific pri-RNA degradation).

### Functional assignment of active miRNAs within miR-371–373 implies conservation of most, if not all, miR-290–295 seeds

Reporter silencing by miR-371–373 could be studied in human embryonic ES cells. However, the miR-371–373 cluster is expressed at much lower levels in human ES cells than miR-290–295 is expressed in mouse ES cells [12], [16], [27]. In fact miR-371–373 expression appears to differ greatly between individual human ES cell lines and/or depend on culture conditions as some studies imply that the cluster is not expressed at all [34], [35]. Thus, it is not *a priori* clear if miR-371–373 reporter silencing in human ES cells would be sufficiently robust for the purposes of this study. Given the quantitative rescue of reporter silencing by transfection of miR-290–295 expression constructs in miR-290–295 null mouse ES cells we reasoned that the shortest route to determining which strands of pre-miR-371–373 yield active miRNAs would be via their heterologous overexpression in mouse ES cells. Co-transfection of miR-371–373 reporters harboring perfectly complementary miRNA binding sites and a miR-371–373 expression construct into miR-290–295 null ES cells resulted in efficient silencing of the miR-371-5p, miR-371-3p, miR-372-3p and miR-373-3p luciferase reporters but not of the reporter constructs carrying miR-372-5p and miR-373-5p target sites (Figure 3D, Figure S3B, Table S1). As with miR-290–295, dilution of the miR-371-373 reporter constructs had no effect on the relative silencing of the reporters confirming that the failure to silence the miR-372-5p and miR-373-5p reporters is not due to excess of the mRNA targets over the hypothetical miRNAs. The assignment of active miRNAs to the 3p- strands of the pre-miR-372 and pre-miR-373 hairpin stems is consistent with the corresponding strand bias in sequencing data from human ES cells (Compare Figures 2 and 3D). However, the silencing of the miR-371-3p reporter is surprising given that most sequencing reads originate from the 5p- strand of the corresponding pre-miRNA stem-loop. Thus, we ruled out the possibility that the miR-371-3p reporter is silenced by miR-372-3p or miR-373-3p by documenting its silencing by a single pre-miR-371 expression construct but not by a rescue construct in which pre-miR-371 was deleted (Figure 3E).

Sequencing data suggest that pre-miR-295 and pre-miR-372 might produce additional 3p+1 isoforms (Figure 2). Such isoforms have identical 6mer 3-7U seeds, but their 7mer seeds are different (3–7A for miR-295-3p+1 and 3-7U for miR-372-3p+1, Figure 1A, Figure S2). Given that the corresponding position 8 is not well conserved in the pre-miR-290–295/pre-miR-371–373 cluster family, 7mer seed target recognition by such 3p+1 isoforms would have to be species specific (Figure 1A, Figure S2). Alternatively, the 3p+1 RNA species implied by the sequencing data might be an artifact that does not represent any active miRNAs. In this case pre-miR-295 and pre-miR-372 would express the same single 2-7U 7mer seed, which is shared with pre-miR-291a, pre-miR-294 and pre-miR373 and, importantly, is represented in all homologous miR-290–295/miR-371–373 loci.

The multiple sequence alignments of pre-miR-290–295/pre-miR-371–373 suggest that pre-miR-371 in the human cluster is capable of producing isomiRs with seeds corresponding to the mouse miR-292-3p0, miR-292-3p+1 and miR-293-3p+2, implied by the sequencing data. The discovery that pre-miR-371 yields active miR-371-3p species in the heterologous mouse system suggests that this might be indeed the case.

Thus, the silencing of perfectly complementary target sites strongly suggests that despite their different pre-miRNA organization the miR-290–295 and miR-371–373 clusters have very similar if not identical seed repertoires. To test this hypothesis we designed reporters that can discriminate between specific miRNA isoforms and their corresponding seeds including overlapping miRNA species processed from the same strand of the same pre-miRNA hairpin stem.

### Seed-specific reporters detect human miR-371-3p+1 and miR-371-3p+2 but rule out miR-371-3p0

One strategy for the functional validation of proposed miRNA isoforms within miR-290–295 and miR-371–373 consists of mutating the seed regions of the perfectly complementary target sites described above with the expectation that mutations, which disrupt pairing of a single miRNA isoform to the target would be less deleterious than mutations, which interfere with the pairing of multiple overlapping miRNA isoforms produced from the same pre-miRNA. This strategy, however, yielded results that are difficult to interpret primarily because position dependent effects seem to outweigh the contribution of individual overlapping miRNA isoforms (Figure S4). This conclusion is consistent with studies, which show that for otherwise perfectly complementary target sites mismatches closer to the 5′-end of the miRNA disrupt silencing less than mismatches that are closer to the middle of the seed region [9].

**Figure 4:**
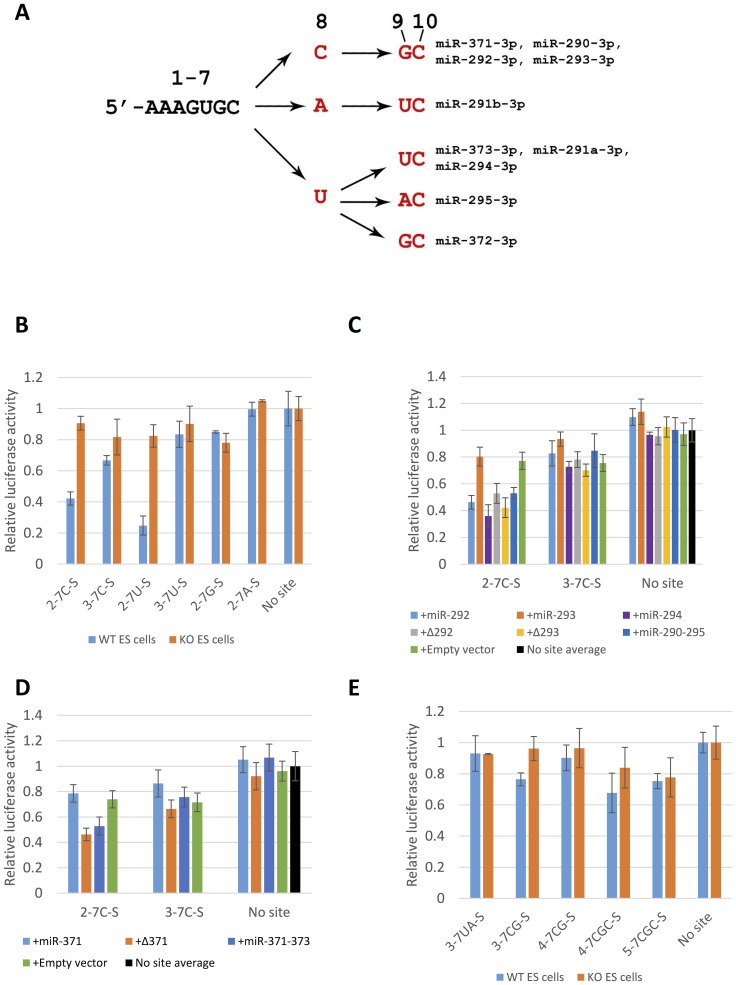
Silencing of seed only miR-290–295/miR-371–373 reporters. (**A**) Sequence variation between multiple sequence alignment positions 3p0 and 3p+9 in pre-miR-290–295/pre-miR-371–373. This panel should be used as key to the various reporter designations. 3p0 miRNA positions 1–7 (equivalent to alignment positions 3p0-3p+7) are invariant in all pre-miRNAs as is 3p0 miRNA position 10 (alignment position 3p+9). Mature miRNAs that correspond to the sequence paths in the graph are shown on the right. (**B**) Silencing of seed only reporters matching all possible miR-290–295/miR-371–373 3p0 miRNA 7mer seeds (2-7C-S, 2-7U-S, 2-7G-S and 2-7A-S) and their position 2 mismatch controls (3-7C-S and 3-7U-S) in wild type (WT) and miR-290–295 knockout (KO) mouse ES cells. (**C, D**) The miR-292-3p0 7mer seed reporter 2-7C-S and its position 2 mismatch control (3-7C-S) were co-transfected into miR-290–295 null ES cells as in Figure 3C, E. +miR-294 is a rescue construct driving a single pre-miR-294 hairpin. (**E**) Silencing of reporters with sequences matching the 7mer seeds of miR-292-3p+1 (3-7CG-S), miR-293-3p+2 (4-7CGC-S) and miR-295-3p+1 (3-7UA-S) and their corresponding seed position 2 mismatch controls (4-7CG-S and 5-7CGC-S) in wild type and miR-290-295 knockout ES cells.

Given the above findings, we reasoned that reporters containing target sites that pair only to the seed regions of miR-290–295/miR-371–373 (seed only targets) or to the seed regions as well as the 3′- regions of the miRNAs but not to the middle of the miRNAs (bubble mismatch targets) might respond to seed mismatches in a more qualitative manner. Because such target sites are not expected to function via Ago2-mediated destabilization, to achieve robust silencing we incorporated four identical tandem target sites in each reporter [30], [31]. Reporter nomenclature follows the notation introduced above for the miR-290–293/miR-371–373 seeds and Figure 4A can be used to track the miRNA - seed interactions detected by the various reporters.

To investigate silencing by the 3p0 miRNAs we generated seed only reporters, which contain sequences complementary to the conserved positions 2–7 of the 3p0 seeds followed by all possible bases at position 8 (Figure 4B, 2–7A,G,C,U-S targets, Table S1). Of these reporters, only 2-7C-S and 2-7U-S were silenced in mouse J1 ES cells, consistent with the predicted absence of isomiRs with 2-7A and 2-7G seeds (Figure 4A, no 3p0 miRNAs within miR-290-295 have a G at position 8 and the 2-7A seed is only present in miR-291b-3p0, which is inactive according to the sequencing and luciferase data). Thus, in our system silencing requires perfect complementarity between the target and the 7mer seed, including pairing to position 8 of the 3p0 miRNAs. Importantly, mutations that disrupt pairing with position 2 of the predicted 3p0 miRNAs strongly interfered or completely abolished silencing confirming specific interactions with the miRNA seeds (Figure 4B, 3-7C-S and 3-7U-S targets).

Surprisingly, rescue experiments in miR-290-295 knockout ES cells revealed that the silencing of the 2-7C-S reporter was not absolutely dependent on the presence of the pre-miR-292 hairpin (Figure 4C, +Δ292, rescue with an expression vector lacking pre-miR-292). Nevertheless, co-transfection of a construct consisting of a single pre-miR-292 hairpin resulted in efficient silencing of the 2-7C-S reporter suggesting that this hairpin does in fact produce an active miR-292-3p0 isoform (Figure 4C, +miR-292 rescue). The 2-7C-S miRNA binding site might be recognized by 3p0 miRNAs with 2-7U seeds (miR-291a-3p0, miR-294-3p0 and miR-295-3p0, Figure 4A) via a G:U wobble at position 8 of the miRNA seed. Indeed, expression of miR-294 in miR-290–295 knockout ES cells resulted in robust silencing of the 2-7C-S reporter, but not of the 3-7C-S reporter (Figure 4C, +miR-294 rescue) strongly suggesting interactions with positions 2–8 of miR-294-3p0. G:U wobbles within the 6mer seed sequence disrupt miRNA-target interactions, but their effect on pairing at position 8 has not been specifically addressed [8], [9].

Co-transfection of a miR-371–373 rescue construct into miR-290–295 knockout ES cells resulted in silencing of the 2-7C-S reporter (Figure 4D). Deletion of pre-miR-371, however, had no effect on silencing (Figure 4D, +Δ371). Thus, similarly to its interaction with the mouse miR-291-3p0, miR-294-3p0 and/or miR-295-3p0, the 2-7C-S target appears to interact via G:U wobble base pairing with miR-372-3p0 and/or miR-373-3p0 which also have a 2-7U seed. However, unlike its putative mouse homolog pre-miR-292, pre-miR-371 alone did not silence the 2-7C reporter (Figure 4D, +miR-371). Thus, pre-miR-371 does not produce an active miR-371-3p0/2-7C seed miRNA.

**Figure 5:**
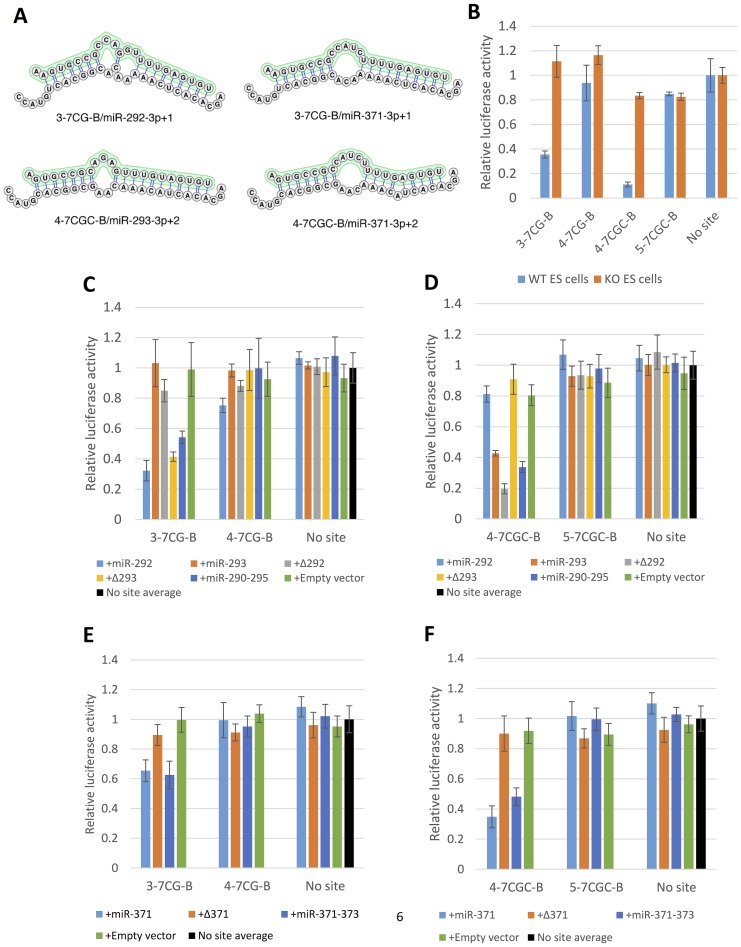
Silencing of bulge-mismatch reporters specific for the 3p+1 and 3p+2 seeds in miR-290–295/miR-371–373. (**A**) Predicted secondary structures of the duplexes formed between miR-292-3p+1 and miR-371-3p+1 with the 3-7CG-B target and of miR-293-3p+2 and miR-371-3p+2 with the 4-7CGC-B target. The miRNA sequences are highlighted. (**B**) Silencing of the miR-292-3p+1 bulge reporter (3-7CG-B) and its position 2 mismatch control (4-7CG-B) and the miR-293-3p+2 bulge reporter (4-7CGC-B) and its wild type mismatch control (5-7CGC-B) in wild type and miR-290-295 knockout ES cells (WT ES cells, KO ES cells). The miR-292-3p+1/miR-371-3p+1 bulge reporter 3-7CG-B and its corresponding position 2 mismatch control 4-7CG-B (**C, E**) or the miR-293-3p+2/miR-371-3p+2 bulge reporter 4-7CGC-B and its position 2 mismatch control 5-7CGC-B (**D, F**) were co-transfected as in Figure 3C, E.

The incorporation of 3-7CG-S, 4-7CGC-S and 3-7UA-S seed only target sites, designed to interact with miR-292-3p+1, miR-293-3p+2 and miR-295-3p+1 respectively resulted in either no silencing or very inefficient silencing of the reporters (Figure 4E). However, the corresponding bulge mismatch reporters 3-7CG-B and 4-7CGC-B were strongly silenced in wild type mouse ES cells (Figure 5A, B). Thus, some feature of the 3-7CG and 4-7CGC seeds make their pairing to the target inefficient but is compensated by increased complementarity between the target and the 3′-portion of the miRNA [9], [31]. Mutations at position 2 of the seed strongly interfered with silencing of the bulge reporters confirming their seed specificity (reporters 4-7CG-B and 5-7CGC-B, Figure 5B). Thus, 3-7CG-B is specifically silenced by a 3p+1 miRNA isoform and 4-7CGC-B is silenced by a 3p+2 miRNA isoform. Importantly, the inefficient silencing of the 4-7CG-B reporter demonstrates that 6mer seed (positions 2–7) pairing of any putative 3p+2 isoforms does not contribute significantly to the silencing of 7mer target 3-7CG-B. Rescue experiments in miR-290–295 knockout ES cells confirmed that silencing of the 3-7CG-B reporter depends on pre-miR-292 and silencing of the 4-7CGC-B reporter depends on pre-miR-293 (Figure 5C, D). Thus, silencing of the bulge reporters proves that active miR-292-3p+1 and miR-293-3p+2 isoforms are expressed in mouse ES cells.

We used the 3-7CG-B and 4-7CGC-B reporters described above to test if the human pre-miR-371 produces 3p+1 and 3p+2 isoforms “seed equivalent” to the mouse miR-292-3p+1 and miR-293-3p+2. Because the sequences of pre-miR-371 on the one hand and pre-miR-292/pre-miR293 on the other vary, the predicted secondary structures of the complexes formed between 3-7CG-B and 4-7CGC-B and their cognate human and mouse miRNAs differ (Figure 5A). Nevertheless, the complexes with miR-371-3p+1 and miR-371-3p+2 preserve the bulge mismatches as well as a high degree of complementarity between the target sequence and the 3′-ends of the miRNAs. Thus, the 3-7CG-B and 4-7CGC-B reporters should be specific for the corresponding miR-371-3p isoforms. Indeed, co-transfection of a miR-371–373 rescue construct into miR-290–295 knockout ES cells resulted in silencing of the 3-7CG-B and 4-7CGC-B reporters (Figure 5E, F). Deletion of pre-miR-371 completely abolished silencing of the 3-7CG-B and 4-7CGC-B reporters and, conversely the pre-miR-371 hairpin alone was able to silence these reporters (Figure 5E, F, +Δ371, +miR-371). Finally, mismatches at position 2 of the bulge reporter seed regions completely abolished silencing confirming interaction with 3p+1 and 3p+2 miRNAs processed from pre-miR-371 (Figure5E, F, 4-7GC-B and 5-7GCG-B reporters).

In summary, the 7mer seed only and bulge reporters confirm the conservation of the 3-7CG and 4-7CGC seeds between the mouse and human clusters and identify the 2-7C seed as unique to the mouse cluster. The active seeds in miR-290–295 and miR-371–373 and the corresponding miRNA isoforms identified in this study are summarized in Table 1.

**Table 1:** Active seeds in miR-290–295 and miR-371–373.

Seed sequence	Code	Mouse miRNAs	Human miRNAs
CUCAAAC	(5p)2-7C	miR-290-5p, miR-292-5p	miR-371-5p
AAGUGCC	(3p)2-7C	miR-292-3p0	-
AGUGCCG	(3p)3-7CG	miR-292-3p+1	miR-371-3p+1
GUGCCGC	(3p)4-7CGC	miR-293-3p+2	miR-371-3p+2
AAGUGCU	(3p)2-7U	miR-291a-3p0, miR-294-3p0, miR-295-3p0	miR-372-3p0, miR-373-3p0
AGUGCUA^a^	(3p)3-7UA?	miR-295-3p+1	-
AGUGCUG^a^	(3p)3-7UG?	-	miR-372-3p+1

a These seeds are implied by the sequencing data, but their activity was not confirmed by reporter silencing in this study.

## Discussion

### Functional equivalence of miR-290–295 and miR-371–373

Our results demonstrate that despite their different pre-miRNA organization the seed repertoires of miR-290–295 in the mouse and miR-371–373 in human are very similar if not identical (Figure 1A, Table 1).

Only two active miRNAs, miR-290-5p0 and miR-292-5p0, which share the same (5p)2-7C seed, are processed from the 5p- strands of the pre-miR-290-295 stems in the mouse. This seed is represented in the human cluster by miR-371-5p0. Reporter silencing implies four distinct “active” seeds corresponding to the 3p- miRNAs in the mouse miR-290-295. Three of these “active” seeds - (3p)2-7U, (3p)3-7CG and (3p)4-7CGC - are conserved in the human miR-371–373. The additional 2-7C seed represented by the mouse miR-292-3p0 is not present in the human cluster. However, we demonstrate that the corresponding target sites are efficiently silenced, potentially via G:U wobble base pairing, by miRNAs containing the conserved 2-7U seed. Thus, miR-292-3p0 does not appear to expand the target repertoire of the mouse cluster.

The reporter silencing experiments presented in this study neither confirm nor confidently rule out the existence of the miR-295-3p+1 and miR-372-3p+1 isoforms implied by sequencing as that requires studying the silencing of the corresponding bulge reporters, which we did not pursue. Nevertheless, we note that the 3-7UA-S seed only reporter, which corresponds to the putative miR-295-3p+1 isoform, had the same activity in wild type and miR-290-295 knockout ES cells, whereas silencing of the 3-7CG-S and 4-7CGC-S reporters, which correspond to the active miR-292-3p+1 and miR-293-3p+2 isomiRs was consistently lower in the wild type ES cells (Figure 4E). This observation is consistent with absence of miR-295-3p+1 activity. The 7mer seed regions that correspond to the putative miR-295-3p+1 and miR-372-3p+1 isoforms are not conserved in the miR-290–295/miR-371-373 cluster family (Figure 1A). Furthermore, as discussed below, pre-miR-295 and pre-miR-372 do not appear to be *bona fide* orthologs. Thus, given the conservation of all other miR-290–295/miR-371–373 seeds we favor a model in which both miR-295-3p+1 and miR-373-3p+1 are not active. If this is indeed the case then the targeting properties of the seven pre-miRNA miR-290–295 and the three pre-miRNA miR-371–373 are not just similar but identical and the two clusters are functionally equivalent.

### pre-miR-290, pre-miR-292 and pre-miR293 are co-orthologs of pre-miR-371: evolution of miR-290-295 from a three-hairpin ancestor

Our data strongly suggest that the mouse pre-miR-290, pre-miR-292 and pre-miR-293 have taken over specialized functions from the single human pre-miR-371 hairpin. pre-miR-371 generates three active miRNA isoforms – miR-371-5p0, miR-371-3p+1 and miR-371-3p+2. In the mouse, their seeds are represented by miR-290-5p0/miR-292-5p0, miR-292-3p+1 and miR-293-3p+2 respectively (Figure 1A, Table 1). With the caveat that miR-295-3p+1 and miR-372-3p+1 might perform species specific functions, the remainder of the miR-290-295/miR-371-373 pre-miRNAs appear to be functionally equivalent since they only produce mature 3p0 miRNAs with identical 2-7U seeds. This proposition is supported by the fact that pre-miR-372 and pre-miR-373, but not pre-miR-371 have oncogenic properties in tissue culture models [21]. The pre-miRNA relationships deduced from the seed expression data are also consistent with the clustering of pre-miR-371, pre-miR-290, pre-miR-292 and pre-miR-293 in a separate branch of the multiple sequence alignment UPMGA tree (Figure 1A).

**Figure 6:**
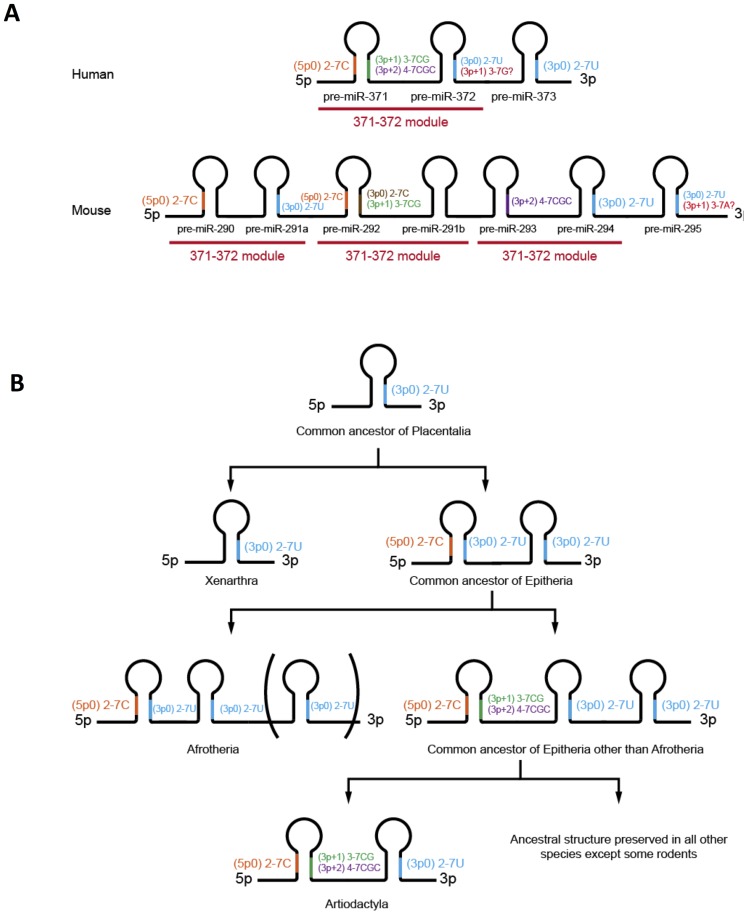
Evolutionary relationships in the miR-290–295/miR-371–373 cluster family. (**A**) Functional homologies between the miR-290–295 and miR-371–373 pre-miRNAs. Schematic representations of the miR-371–373 (top) and miR-290–295 (bottom) clusters are shown together with the active miRNA isoforms processed from each pre-miRNA hairpin and their corresponding seeds. The mouse cluster contains three repeats, which are homologous to the miR-371–373 region in human (371–372 module). (**B**) Evolution of the miR-290–295/miR-371–373 cluster family in *Placentalia*. Schematic representations of the miR-290–295/miR-371–373 homologs in placental mammals are shown together with the postulated miRNA isoforms and corresponding seed sequences processed from the individual pre-miRNA hairpins.

In the miR-290-295 cluster the specialized pre-miR-371 co-orthologs are interspaced by pre-miRNAs, which are processed, or in the case of pre-miR-291b could potentially be processed, into isomiRs that contain (3p)2-7U seeds. Thus, the mouse cluster likely evolved from a three pre-miRNA ancestor via duplication of a module consisting of the promoter proximal and middle pre-miRNAs (pre-miR-371 and pre-miR-372 in human, Figure 6A). Therefore, pre-miR-291a, pre-miR-291b and pre-miR294 are likely co-orthologs of pre-miR-372 and pre-miR-295 is an ortholog of the promoter distal pre-miR-373. Note that in this scenario, the putative species-specific miR-295-3p+1 and miR-372-3p+1 isoforms are processed from different paralogous pre-miRNA families.

### Evolution of the miR-290–295/miR-371–373 family in Placentals

The poor phylogenetic signal in the short miRNA and pre-miRNA sequences makes it difficult to reconstruct evolutionary relationships precisely solely by sequence comparisons. The elucidation of the seed repertoires of miR-290–295 and miR-371–373, however, provides additional information that allows the reconstruction of the miR-290–295/miR-371–373 cluster family evolution by focusing on the acquisition of new seeds.

Conservation of the miR-292-3p+1/miR-371-3p+1 and miR-293-3p+2/miR-371-3p+2 seeds requires that the dinucleotide sequence CG is present at positions 3p+7 and 3p+8 of the pre-miRNA multiple sequence alignment (Figure 1A). This dinucleotide is only present in the promoter-proximal pre-miRNAs of the miR-290–295/miR-371–373 cluster family (pre-miR-290 and pre-miR-371 in human) and the additional paralogs in the mouse miR-290–295 (pre-miR-292 and pre-miR-293). Notable exceptions to this rule are the clusters from the three orders of *Afrotheria* where the corresponding sequence is UG (Figure 1A, sequences #38-40). Thus, the corresponding 3p+1 and 3p+2 miRNAs are either not processed in *Afrotheria* or contain non-conserved seeds. Together with the known evolutionary relationships between placental mammals (Figure 1B) [29], this observation and the miR-290-295/miR-371-373 seed conservation data presented here lead to an attractive model for the evolution of the miR-290-295/miR-371-373 cluster family (Figure 6B). We postulate that the single pre-miRNA in *Xenarthra* and the common ancestor of all *Plancetalia* yields only a 3p0 miRNA with a (3p)2-7U seed. Duplication of this single ancestral pre-miRNA together with secondary structure changes in the promoter-proximal pre-miRNA result in the acquisition of a 5p- miRNA with a (5p)2-7C seed in the common ancestor of all *Epitheria* and in descendant *Afrotheria* as well as additional 3p+1 and 3p+2 isomiRs with (3p)3-7CG and (3p+2)4-7CGC seeds in the common ancestor of *Epitheria* other than *Afrotheria* and all corresponding descendant species. In the proposed model, the three pre-miRNA structure of the cluster in *Hyracoidea* and the two-hairpin structure of the cluster in *Artiodactyla* reflect respectively a duplication and a deletion of a (3p)2-7U seed pre-miRNA. Thus, the two-hairpin structure of the miR-290–295/miR-371–373 clusters in *Afrosoricida and Proboscidea* on the one hand and *Artiodactyla* on the other reflects completely different evolutionary events.

### Models of the miR-290–295/miR-371–373 target interaction networks should incorporate the pre-miR-371/pre-miR-290/pre-miR-292/pre-miR-293 seeds

All well-established biological functions of the miR-290–295/miR-371–373 cluster family - regulation of the G1/S transition in ES cells, suppression of apoptosis in ES cells, promoting iPS cell reprogramming, germ cell tumorigenesis and indirect effects on ES cell genome methylation – depend on target suppression via the 2–7 U seed [22]–[26]. The majority of miRNA targets in mouse ES cells identified via HITS-CLIP also pair to the 2–7 U seed [27]. This seed is shared with miRNAs that are otherwise unrelated such as the miR-430, miR-302 and miR-467a families (http://www.mirbase.org [7]). miR-430 and miR-302 appear in the zebrafish and chick genomes and have therefore been acquired before the split of the mammalian lineage. In addition, miR-302 and miR-467a are expressed in mouse ES cells and supplement silencing of the miR-290–295 targets via the 2–7 U seed [16], [27]. The miR-302 cluster is the most abundant miRNA family in human ES cells, and appears to be mostly responsible for 2–7 U seed functions instead of miR-371–373, which is expressed at much lower levels [12]. Finally, the important role that the miR-430 family plays during early zebrafish development implies that interaction networks involving the 2-7U seed are likely conserved in all vertebrates [36]. Thus, present models of miR-290–295/miR-371–373 function focus on relatively ancient miR-290–295/miR-371–373-target interaction networks involving the 2–7 U seed family. Target interaction networks involving the more recently acquired (5p)2-7U, (3p)3-7CG and (3p)4-7CGC miRNA seed families, which exist only in the Epitherian lineage of placental mammals have received little, if any, attention. Their specific loss of function phenotype is presently unknown and the corresponding targets in HITS-CLIP experiments are few and much less statistically robust than those of the 2-7U family [27].

While some recently evolved miRNA seeds are thought not to have had enough evolutionary time to acquire physiologically relevant targets, the conservation of the (5p)2-7U, (3p)3-7CG and (3p)4-7CGC seeds in the miR-290-295/miR-371-373 family, particularly in the seven hairpin mouse miR-290-295 cluster where these seeds are distributed between three separate pre-miRNAs that have otherwise diverged considerably from their ancestral pre-miRNA, strongly suggests that they perform specific functions in developmental aspects that are unique to the Epitherian lineage. Given the involvement of the 2-7U seed in cell proliferation and survival, it is likely that the phenotypes caused by the loss of the 2-7U seed miRNAs in the miR-290-295 knockout mouse mask any specific phenotypes due to the loss of function of pre-miR-290, pre-miR-292 and pre-miR-293 [20]. Furthermore, the lack of high confidence targets for the pre-miR-290/pre-miR-292/pre-miR-293 seeds in HITS-CLIP data from mouse ES cells suggests that the corresponding miRNAs might be physiologically relevant in other biological contexts such as the extraembryonic lineages and/or TS and XEN cells.

The silencing of miR-371-3p reporters in mouse ES cells overexpressing miR-371-373 is at odds with sequencing data, which suggest that miR-371-5p, but not miR-371-3p is expressed in human ES cells. While the miR-371-373 sequencing data is less comprehensive than the miR-290-295 sequencing data and, thus, this discrepancy might be due to amplification bias or some other sequencing library construction artifact, it is tempting to speculate that miR-371-3p processing or loading into miRISC might be differentially regulated in human and mouse ES cells, which likely represent different compartments of the mammalian embryo [37]–[39]. If this hypothesis is correct, then processing of the corresponding mouse pre-miR-290, pre-miR-292 and pre-miR-293 might also be differentially regulated.

## Conclusions

A hallmark feature of the evolution of the miR-290–295/miR-371–373 cluster family is the gradual addition of new miRNA seeds and it is tempting to speculate that the built in propensity of these clusters to generate co-expressed multiple distinct seeds reflects some undiscovered general property of the corresponding target interaction networks. Short RNA sequencing data mining, seed specific miRNA reporter studies and experimental as well as computational approaches for miRNA cluster target identification can be combined to test this idea.

## Materials and Methods

### Bioinformatics

Multiple sequence alignments and UPMGA tree assignments were performed with CLUSTALW [40] and Geneious (http://www.geneious.com). BLAST searches were done online via the ENSEMBL server (http://www.ensembl.org). HMMER searches were performed locally under MacOS X [28]. RNA secondary structures were computed with the RNAfold and RNAcofold utilities of the ViennaRNA package and visualized with the VARNA Java applet [41], [42]. Short RNA sequencing data was processed with custom PERL and C utilities.

### Cell lines and tissue culture

J1 and miR-290–295 ES cells were a gift from Rudolf Jaenisch and Phillip Sharp and were propagated by standard protocols [20], [26], [43], [44]. The cells were grown in DMEM with 15% fetal calf serum and 1000 u/mL leukemia inhibitory factor (ESGRO, Millipore) on gelatinized plastic and in the absence of feeders. Transfections were performed with Lipofectamine 2000 (Invitrogen) according to the protocols supplied by the manufacturer.

### Plasmids

All plasmids in this study are derivatives of pArgoN in which the gene of interest is under the control of the synthetic CAG promoter [32]. pArgoP contains a synthetic oligonucleotide insertion into the NotI site of pArgoN to generate a polylinker. The Renilla and firefly luciferase genes were subcloned from pRL-TK and pGL3-basic plasmids (Promega) into pArgoN yielding pArg-RL and pArg-FF. pArg-FF-P has the polylinker of pArgoP added to pArg-FF. miRNA target sites were inserted into pArg-FF (perfectly complementary miR-290–295 targets) or pArg-FF-P (perfectly complementary miR-371–373 targets, seed only targets and bulge targets) as synthetic oligonucleotides. The miR-290–295 and miR-371–373 expression constructs, pArgF101-290–295 and pArgF101-371–373, consist of PCR amplified BAC fragments inserted into the pArgoP derivative pArgF101+, which has an added polyomavirus origin of replication [45]. The annotated sequences of pArgoN, pArgoP, pArg-FF, pArg-FF-P, pArg-RL and pArgF101+ are given as supplementary data and detailed information about their construction is available upon request. Additional details can be found in the supplementary material.

### Reporter assays

Firefly and Renilla luciferase assays were performed with the Dual Luciferase Assay kit and a Glomax 20/20 dual injector luminometer (Promega) according to the protocols supplied by the manufacturer. Cells were harvested 24–48 hours after the transfection. Reporter activities are expressed as the ratio of the firefly and Renilla luciferase activities and are normalized to the no-target control. When multiple independently performed experiments are merged into one figure normalization is to the average of the no-target controls from the different experiments.

## Supporting Information

Figure S1 **Short RNA 5′-end distributions for pre-miR-291b.** Normalized frequencies of the 5′-end positions of RNA species that map to pre-miR-291b in various sequencing datasets. See the legend to Figure 2.
(TIF)

Figure S2 **Multiple sequence alignment of the perfectly complementary target sites, in miRNA sense orientation (** **Table 1** **), designed to detect 5p (A) and 3p (B) miRNAs processed from miR-290–295 and miR-371–373.** The oligonucleotide sequences correspond to parts of the pre-miRNAs shown in Figure 1A and the alignment was recomputed.
(TIF)

Figure S3 **Additional dilutions of the experiments shown in** **Figure 3C, D** **.** The reporters could not be diluted any further as that resulted in background luciferase activity.
(TIF)

Figure S4 **Mismatches to the indicated positions of hypothetical miR-292-5p0, miR-292-3p0, miR-293-3p0, miR-294-3p0 and miR-295-3p0 species were introduced into the corresponding 292-5p-5p, 292-3p-P, 293-3p-P, 294-3p-P and 295-3p-P perfectly complementary reporters and their activities were measured by luciferase assays.** The mismatches are labeled according to the isomiR nomenclature explained in the text.
(TIF)

Table S1 **Sequences of the miRNA target sites used in this study.**
(XLSX)

Dataset S1 **Supplementary Luciferase Assays Raw Data.**
(XLSX)

Dataset S2 **Supplementary Luciferase Assays Raw Data.**
(XLSX)

Dataset S3 **Supplementary Luciferase Assays Raw Data.**
(XLSX)

Dataset S4 **Supplementary Luciferase Assays Raw Data.**
(XLSX)

Dataset S5 **Supplementary Luciferase Assays Raw Data.**
(XLSX)

File S1 **Supplementary Materials and Methods.doc: Detailed experimental procedures.**
(DOCX)

Sequence S1 **Annotated plasmid sequences in GenBank format: sequence of plasmid pArg-FF-P.**
(GB) Click here for additional data file.

Sequence S2 **Annotated plasmid sequences in GenBank format: sequence of plasmid pArg-RL.**
(GB) Click here for additional data file.

Sequence S3 **Annotated plasmid sequences in GenBank format: sequence of plasmid pArgoN.**
(GB) Click here for additional data file.

Sequence S4 **Annotated plasmid sequences in GenBank format: sequence of plasmid pArgoP.**
(GB) Click here for additional data file.

Sequence S5 **Annotated plasmid sequences in GenBank format: sequence of plasmid PF101+.**
(GB) Click here for additional data file.

## References

[1] Berezikov E (2011) Evolution of microRNA diversity and regulation in animals. Nat Rev Genet 12: 846–860. 22094948 10.1038/nrg3079

[2] Campo-Paysaa F, Semon M, Cameron RA, Peterson KJ, Schubert M (2011) microRNA complements in deuterostomes: origin and evolution of microRNAs. Evol Dev 13: 15–27. 21210939 10.1111/j.1525-142X.2010.00452.x

[3] Griffiths-Jones S, Hui JH, Marco A, Ronshaugen M (2011) MicroRNA evolution by arm switching. EMBO Rep 12: 172–177. 21212805 10.1038/embor.2010.191 PMC3049427

[4] Lau NC, Lim LP, Weinstein EG, Bartel DP (2001) An abundant class of tiny RNAs with probable regulatory roles in Caenorhabditis elegans. Science 294: 858–862. 11679671 10.1126/science.1065062

[5] Lagos-Quintana M, Rauhut R, Lendeckel W, Tuschl T (2001) Identification of novel genes coding for small expressed RNAs. Science 294: 853–858. 11679670 10.1126/science.1064921

[6] Chiang HR, Schoenfeld LW, Ruby JG, Auyeung VC, Spies N, et al (2010) Mammalian microRNAs: experimental evaluation of novel and previously annotated genes. Genes Dev 24: 992–1009. 20413612 10.1101/gad.1884710 PMC2867214

[7] Griffiths-Jones S (2004) The microRNA Registry. Nucleic Acids Res 32 Database issue: D109–11. 10.1093/nar/gkh023 PMC308757 14681370

[8] Doench JG, Sharp PA (2004) Specificity of microRNA target selection in translational repression. Genes Dev 18: 504–511. 15014042 10.1101/gad.1184404 PMC374233

[9] Wee LM, Flores-Jasso CF, Salomon WE, Zamore PD (2012) Argonaute divides its RNA guide into domains with distinct functions and RNA-binding properties. Cell 151: 1055–1067. 23178124 10.1016/j.cell.2012.10.036 PMC3595543

[10] Schwarz DS, Hutvagner G, Du T, Xu Z, Aronin N, et al (2003) Asymmetry in the assembly of the RNAi enzyme complex. Cell 115: 199–208. 14567917 10.1016/s0092-8674(03)00759-1

[11] Fukunaga R, Han BW, Hung JH, Xu J, Weng Z, et al (2012) Dicer partner proteins tune the length of mature miRNAs in flies and mammals. Cell 151: 533–546. 23063653 10.1016/j.cell.2012.09.027 PMC3609031

[12] Morin RD, O'Connor MD, Griffith M, Kuchenbauer F, Delaney A, et al (2008) Application of massively parallel sequencing to microRNA profiling and discovery in human embryonic stem cells. Genome Res 18: 610–621. 18285502 10.1101/gr.7179508 PMC2279248

[13] Houbaviy HB, Murray MF, Sharp PA (2003) Embryonic stem cell-specific MicroRNAs. Dev Cell 5: 351–358. 12919684 10.1016/s1534-5807(03)00227-2

[14] Suh MR, Lee Y, Kim JY, Kim SK, Moon SH, et al (2004) Human embryonic stem cells express a unique set of microRNAs. Dev Biol 270: 488–498. 15183728 10.1016/j.ydbio.2004.02.019

[15] Houbaviy HB, Dennis L, Jaenisch R, Sharp PA (2005) Characterization of a highly variable eutherian microRNA gene. Rna 11: 1245–1257. 15987809 10.1261/rna.2890305 PMC1370808

[16] Calabrese JM, Seila AC, Yeo GW, Sharp PA (2007) RNA sequence analysis defines Dicer's role in mouse embryonic stem cells. Proc Natl Acad Sci U S A 104: 18097–18102. 17989215 10.1073/pnas.0709193104 PMC2084302

[17] Spruce T, Pernaute B, Di-Gregorio A, Cobb BS, Merkenschlager M, et al (2010) An early developmental role for miRNAs in the maintenance of extraembryonic stem cells in the mouse embryo. Dev Cell 19: 207–219. 20708584 10.1016/j.devcel.2010.07.014

[18] Tang F, Kaneda M, O'Carroll D, Hajkova P, Barton SC, et al (2007) Maternal microRNAs are essential for mouse zygotic development. Genes Dev 21: 644–648. 17369397 10.1101/gad.418707 PMC1820938

[19] Hayashi K, Chuva de Sousa Lopes SM, Kaneda M, Tang F, Hajkova P, et al (2008) MicroRNA biogenesis is required for mouse primordial germ cell development and spermatogenesis. PLoS ONE 3: e1738. 18320056 10.1371/journal.pone.0001738 PMC2254191

[20] Medeiros LA, Dennis LM, Gill ME, Houbaviy H, Markoulaki S, et al (2011) Mir-290–295 deficiency in mice results in partially penetrant embryonic lethality and germ cell defects. Proc Natl Acad Sci U S A 108: 14163–14168. 21844366 10.1073/pnas.1111241108 PMC3161528

[21] Voorhoeve PM, le Sage C, Schrier M, Gillis AJ, Stoop H, et al (2006) A genetic screen implicates miRNA-372 and miRNA-373 as oncogenes in testicular germ cell tumors. Cell 124: 1169–1181. 16564011 10.1016/j.cell.2006.02.037

[22] Wang Y, Baskerville S, Shenoy A, Babiarz JE, Baehner L, et al (2008) Embryonic stem cell-specific microRNAs regulate the G1-S transition and promote rapid proliferation. Nat Genet 40: 1478–1483. 18978791 10.1038/ng.250 PMC2630798

[23] Judson RL, Babiarz JE, Venere M, Blelloch R (2009) Embryonic stem cell-specific microRNAs promote induced pluripotency. Nat Biotechnol. 10.1038/nbt.1535 PMC2743930 19363475

[24] Subramanyam D, Lamouille S, Judson RL, Liu JY, Bucay N, et al (2011) Multiple targets of miR-302 and miR-372 promote reprogramming of human fibroblasts to induced pluripotent stem cells. Nat Biotechnol 29: 443–448. 21490602 10.1038/nbt.1862 PMC3685579

[25] Sinkkonen L, Hugenschmidt T, Berninger P, Gaidatzis D, Mohn F, et al (2008) MicroRNAs control de novo DNA methylation through regulation of transcriptional repressors in mouse embryonic stem cells. Nat Struct Mol Biol 15: 259–267. 18311153 10.1038/nsmb.1391

[26] Zheng GX, Ravi A, Calabrese JM, Medeiros LA, Kirak O, et al (2011) A latent pro-survival function for the mir-290–295 cluster in mouse embryonic stem cells. PLoS Genet 7: e1002054. 21573140 10.1371/journal.pgen.1002054 PMC3088722

[27] Leung AK, Young AG, Bhutkar A, Zheng GX, Bosson AD, et al (2011) Genome-wide identification of Ago2 binding sites from mouse embryonic stem cells with and without mature microRNAs. Nat Struct Mol Biol 18: 237–244. 21258322 10.1038/nsmb.1991 PMC3078052

[28] Eddy SR, Mitchison G, Durbin R (1995) Maximum discrimination hidden Markov models of sequence consensus. J Comput Biol 2: 9–23. 7497123 10.1089/cmb.1995.2.9

[29] O'Leary MA, Bloch JI, Flynn JJ, Gaudin TJ, Giallombardo A, et al (2013) The placental mammal ancestor and the post-K-Pg radiation of placentals. Science 339: 662–667. 23393258 10.1126/science.1229237

[30] Doench JG, Petersen CP, Sharp PA (2003) siRNAs can function as miRNAs. Genes Dev 17: 438–442. 12600936 10.1101/gad.1064703 PMC195999

[31] Grimson A, Farh KK, Johnston WK, Garrett-Engele P, Lim LP, et al (2007) MicroRNA targeting specificity in mammals: determinants beyond seed pairing. Mol Cell 27: 91–105. 17612493 10.1016/j.molcel.2007.06.017 PMC3800283

[32] Niwa H, Yamamura K, Miyazaki J (1991) Efficient selection for high-expression transfectants with a novel eukaryotic vector. Gene 108: 193–199. 1660837 10.1016/0378-1119(91)90434-d

[33] Mukherji S, Ebert MS, Zheng GX, Tsang JS, Sharp PA, et al (2011) MicroRNAs can generate thresholds in target gene expression. Nat Genet 43: 854–859. 21857679 10.1038/ng.905 PMC3163764

[34] Lipchina I, Elkabetz Y, Hafner M, Sheridan R, Mihailovic A, et al (2011) Genome-wide identification of microRNA targets in human ES cells reveals a role for miR-302 in modulating BMP response. Genes Dev 25: 2173–2186. 22012620 10.1101/gad.17221311 PMC3205587

[35] Bar M, Wyman SK, Fritz BR, Qi J, Garg KS, et al (2008) MicroRNA discovery and profiling in human embryonic stem cells by deep sequencing of small RNA libraries. Stem Cells 26: 2496–2505. 18583537 10.1634/stemcells.2008-0356 PMC2847579

[36] Giraldez AJ, Mishima Y, Rihel J, Grocock RJ, Van Dongen S, et al (2006) Zebrafish MiR-430 promotes deadenylation and clearance of maternal mRNAs. Science 312: 75–79. 16484454 10.1126/science.1122689

[37] Brons IG, Smithers LE, Trotter MW, Rugg-Gunn P, Sun B, et al (2007) Derivation of pluripotent epiblast stem cells from mammalian embryos. Nature 448: 191–195. 17597762 10.1038/nature05950

[38] Tesar PJ, Chenoweth JG, Brook FA, Davies TJ, Evans EP, et al (2007) New cell lines from mouse epiblast share defining features with human embryonic stem cells. Nature 448: 196–199. 17597760 10.1038/nature05972

[39] Pera MF, Tam PP (2010) Extrinsic regulation of pluripotent stem cells. Nature 465: 713–720. 20535200 10.1038/nature09228

[40] Larkin MA, Blackshields G, Brown NP, Chenna R, McGettigan PA, et al (2007) Clustal W and Clustal X version 2.0. Bioinformatics 23: 2947–2948. 17846036 10.1093/bioinformatics/btm404

[41] Lorenz R, Bernhart SH, Honer Zu Siederdissen C, Tafer H, Flamm C, et al (2011) ViennaRNA Package 2.0. Algorithms Mol Biol 6: 26. 22115189 10.1186/1748-7188-6-26 PMC3319429

[42] Darty K, Denise A, Ponty Y (2009) VARNA: Interactive drawing and editing of the RNA secondary structure. Bioinformatics 25: 1974–1975. 19398448 10.1093/bioinformatics/btp250 PMC2712331

[43] Li E, Bestor TH, Jaenisch R (1992) Targeted mutation of the DNA methyltransferase gene results in embryonic lethality. Cell 69: 915–926. 1606615 10.1016/0092-8674(92)90611-f

[44] Nagy A, Gertsenstein M, Vintersten K, Behringer R (2003) Manipulating the mouse embryo. A laboratory manual. Cold Spring Harbor, New York: Cold Spring Harbor Laboratory Press.

[45] Gassmann M, Donoho G, Berg P (1995) Maintenance of an extrachromosomal plasmid vector in mouse embryonic stem cells. Proc Natl Acad Sci U S A 92: 1292–1296. 7877970 10.1073/pnas.92.5.1292 PMC42505

