# Supplementary figures and images for: Evolution of the miR-290–295/miR-371–373 Cluster Family Seed Repertoire
Source: PLoS One. 2014 Sep 30;9(9):e108519. doi: 10.1371/journal.pone.0108519 (PMC4182485; doi:10.1371/journal.pone.0108519)

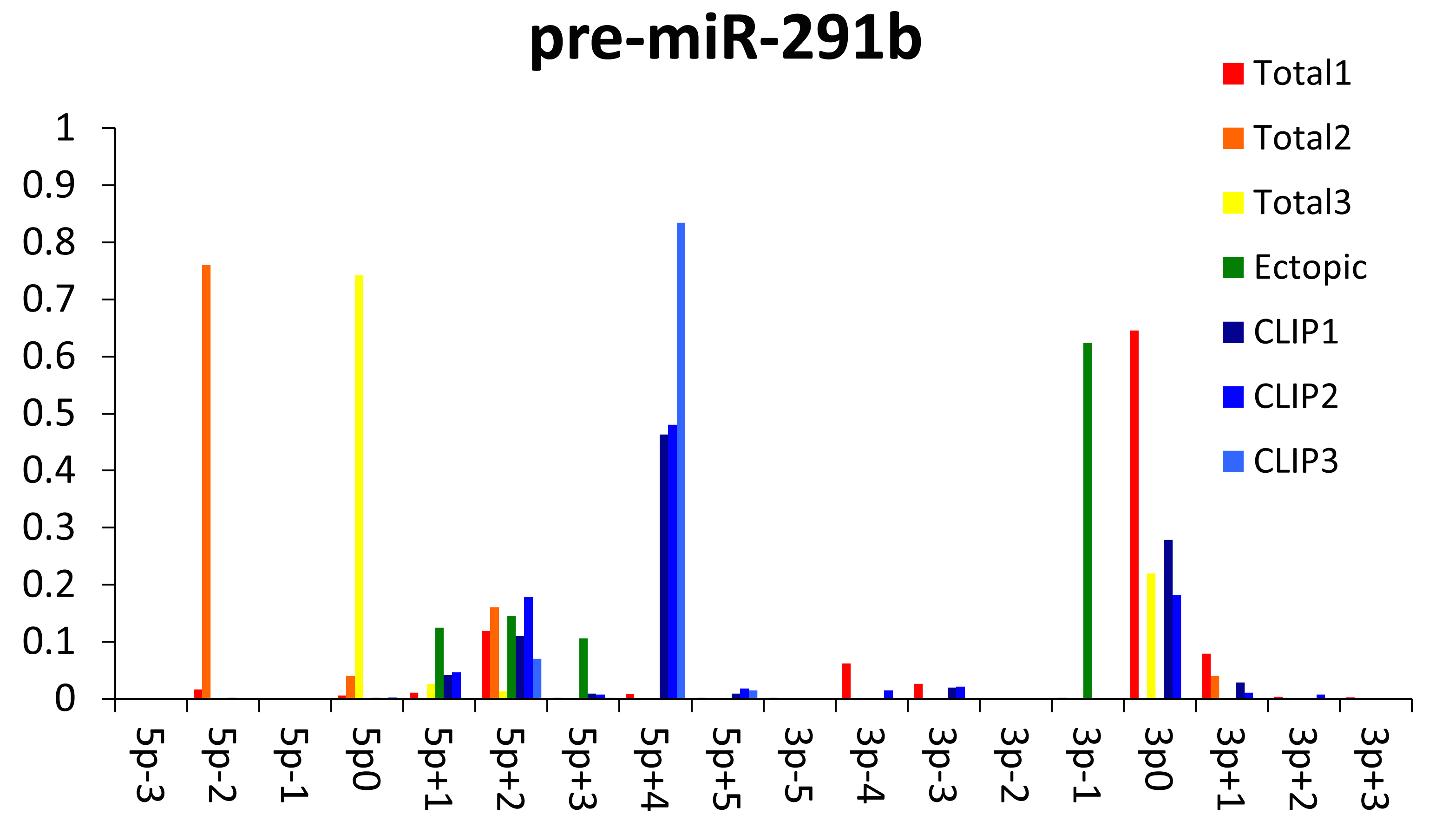

Supplement: Figure S1 — Short RNA 5′-end distributions for pre-miR-291b. Normalized frequencies of the 5′-end positions of RNA species that map to pre-miR-291b in various sequencing datasets. See the legend to Figure 2. (TIF) [file pone.0108519.s001.tif]

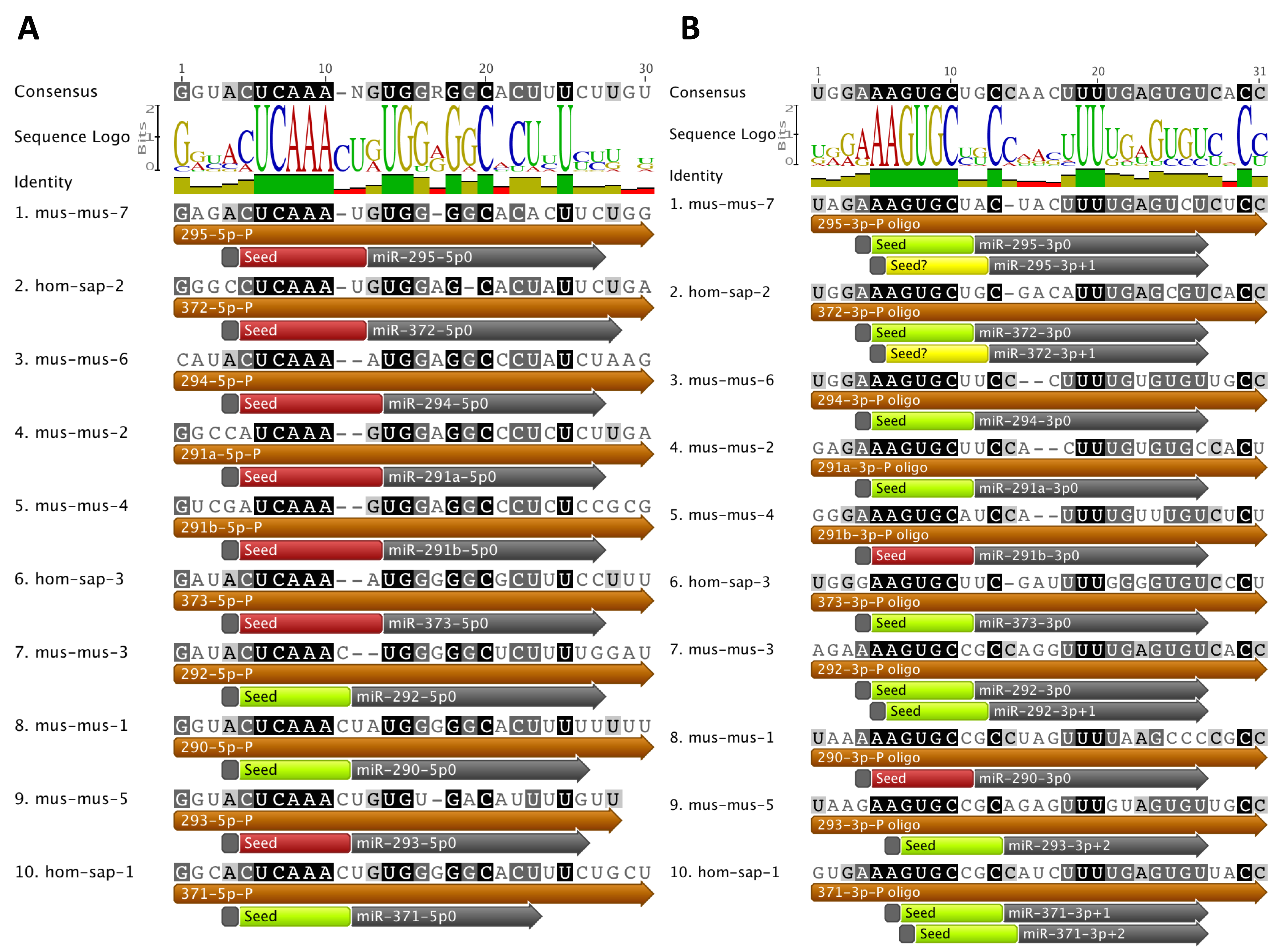

Supplement: Figure S2 — Multiple sequence alignment of the perfectly complementary target sites, in miRNA sense orientation ( Table 1 ), designed to detect 5p (A) and 3p (B) miRNAs processed from miR-290–295 and miR-371–373. The oligonucleotide sequences correspond to parts of the pre-miRNAs shown in Figure 1A and the alignment was recomputed. (TIF) [file pone.0108519.s002.tif]

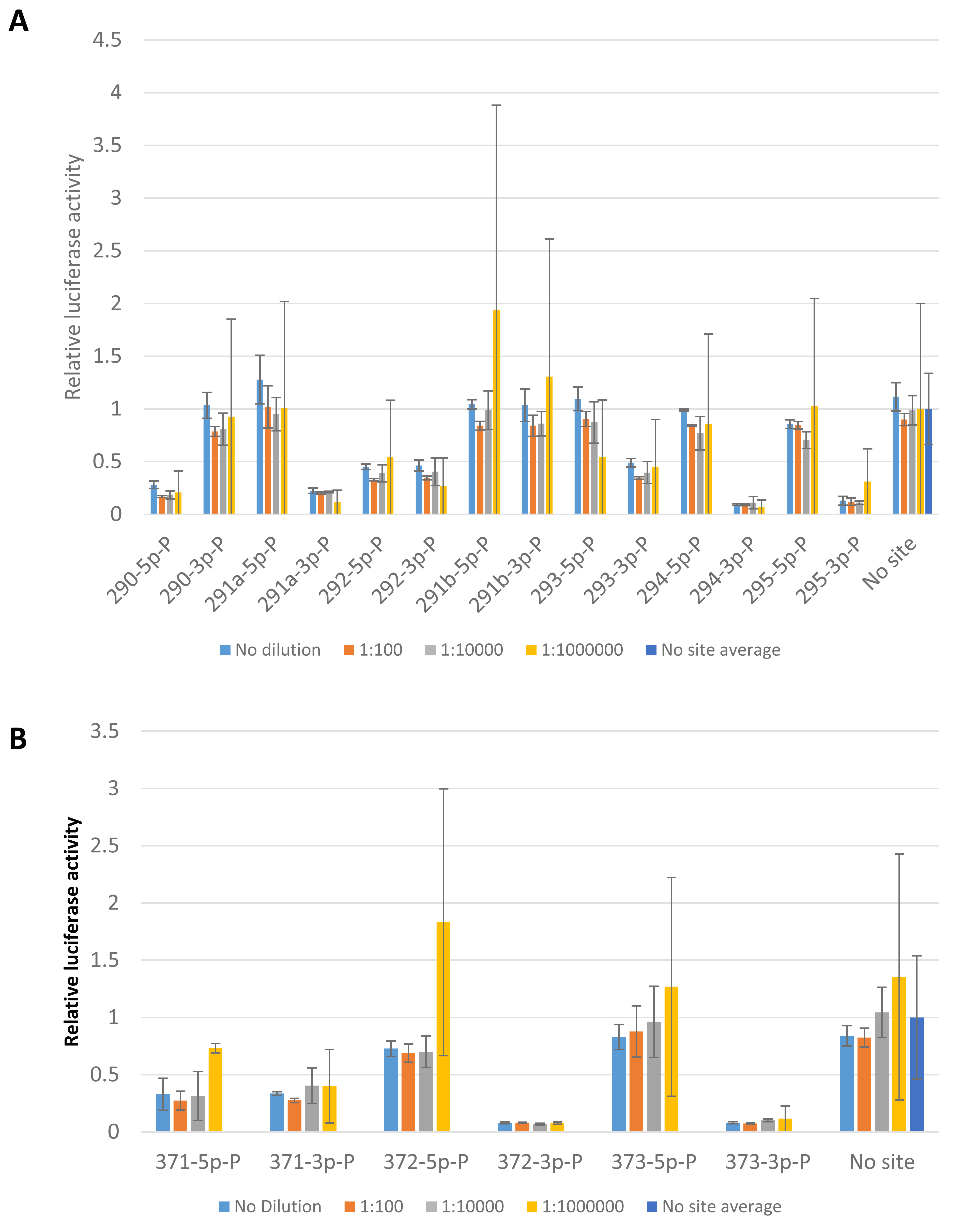

Supplement: Figure S3 — Additional dilutions of the experiments shown in Figure 3C, D . The reporters could not be diluted any further as that resulted in background luciferase activity. (TIF) [file pone.0108519.s003.tif]

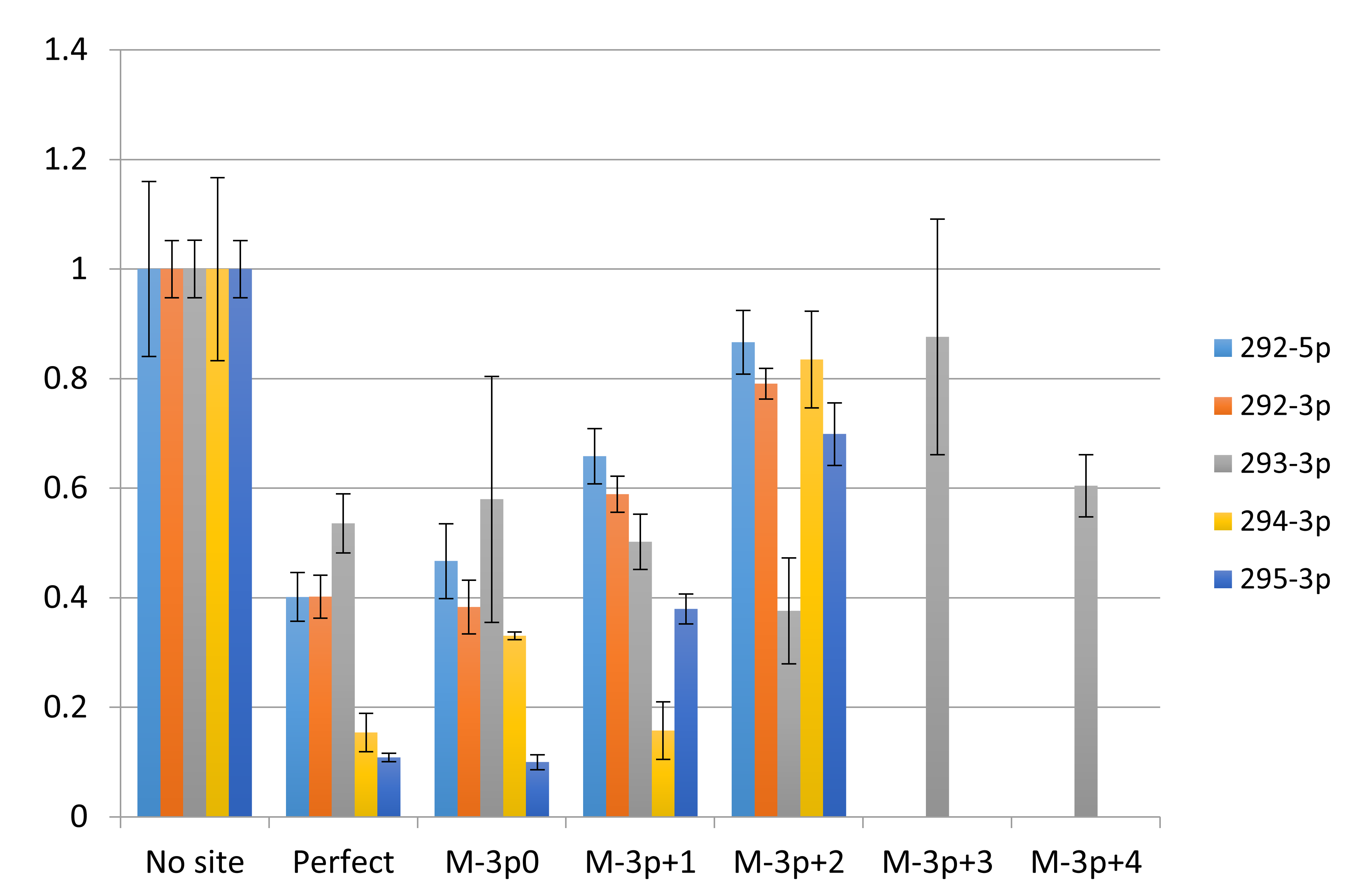

Supplement: Figure S4 — Mismatches to the indicated positions of hypothetical miR-292-5p0, miR-292-3p0, miR-293-3p0, miR-294-3p0 and miR-295-3p0 species were introduced into the corresponding 292-5p-5p, 292-3p-P, 293-3p-P, 294-3p-P and 295-3p-P perfectly complementary reporters and their activities were measured by luciferase assays. The mismatches are labeled according to the isomiR nomenclature explained in the text. (TIF) [file pone.0108519.s004.tif]
