# Supplementary material for: Evolution of the miR-290–295/miR-371–373 Cluster Family Seed Repertoire
Source: PLoS One. 2014 Sep 30;9(9):e108519. doi: 10.1371/journal.pone.0108519 (PMC4182485; doi:10.1371/journal.pone.0108519)
Supplement: File S1 — Supplementary Materials and Methods.doc: Detailed experimental procedures. (DOCX) [file pone.0108519.s011.docx]

**Supplementary Materials and Methods**

**Plasmid construction**

**1. pArgoN derivatives**

pArgoP and pArg-FF-P were derived by inserting synthetic oligonucleotide duplex

F: 5’-GGCCGGATCCGCTAGCGAGCTCGC-3’ (non-template strand of the CAG promoter)

R: 5’-GGCCGCGAGCTCGCTAGCGGATCC-3’ (template strand of the CAG promoter)

into the NotI sites of pArgoN and pArg-FF respectively after destroying the BamHI site adjacent to the PGK promoter by partial BamHI digestion, Klenow fragment re-filling and re-ligation (see GenBank sequence files). The oligonucleotide insertion introduces a BamHI, NheI, SacI polylinker downstream of the CAG promoter/firefly luciferase gene.

pArgF101+ was generated by inserting a BglII digested PCR fragment of pMGD20neo {Gassmann et al., 1995, #2764} containing the PyF101 origin of replication amplified with primers (BlgII sequences highlighted)

F: 5’-GGAAGATCTGGCCAGATCCAGACATGATAAGAT-3’ (BglII)

R: 5’-GGAAGATCTCAGCTCTGCTCAGAACTCTATCCAT-3’ (BglII)

into the BamHI site of pArgoN, followed by insertion of a polylinker as described above. In the resulting construct the early promoter of the PyF101 fragment is in the same orientation as the PGK promoter driving the puromycin resistance gene.

**2. Expression vectors**

**2.1. Full length miR-290-295/miR-371 expression vectors**

The miR-290-295 region was PCR amplified from BAC RP23-222D21 (http://bacpac.chori.org) with primers

F (F): 5’-CGTAGAGCTCTCTCCACGTTCTTTCCTCCTGGT-3’ (SacI)

R (R): 5’-CGTAGCGGCCGCACACCCTGCTCCCCAACCCA-3’ (NotI)

and inserted into the SacI-NotI sites of pArgF101+ to yield pArgF101-miR-290-295.

The miR-371-373 region was PCR amplified from BAC RP11-381E3 with primers

F : 5’-CGTAGGATCCCTTCCACTTGCGATCGCC-3’ (BamHI)

R: 5’-CGTAGCGGCCGCGGAACCCAATAGACCAGAAAC-3’ (NotI)

and inserted into the BamHI-NotI sites of pArgF101+ to yield pArgF101-miR-371-373.

**2.2. Single pre-miRNA expression vectors**

The single hairpin expression constructs consist of PCR amplified fragments of pArgF101-miR-290-295 and pArgF101-miR-371-373 inserted into the SacI – NotI sites of pArgP. The primer pairs are as follows (restriction sites highlighted)

pArg-miR-292:

F: 5’-CGTAGAGCTCTCTGACGCATCGGTTTACAG-3’  (SacI)

R: 5’-CGTAGCGGCCGCCTCCTTTATGAACGCGGAAA-3’ (NotI)

pArg-miR-293:

F: 5’-CGTAGAGCTCGTGGACAGCCGGGTAGATAA-3’ (SacI)

R: 5’-CGTAGCGGCCGCTTCGCTAGATCCAAACATGC-3’ (NotI)

pArg-miR-294:

F: 5’-CGTAGAGCTCTGCAGTTGGCCTAAGTGTTG-3’  (SacI)

R: 5’-CGTAGCGGCCGCCTCACCAAGATGAGCAAGCA-3’  (NotI)

pArg-miR-371:

F: 5’-CGTAGGATCCCTTCCACTTGCGATCGCC-3’  (SacI)

R: 5’-CGTAGCGGCCGCCAGCTCCATCTGCAAGAGC-3’  (NotI)

**2.3. Expression vectors containing single pre-miRNA deletions**

Single pre-miRNA deletions were performed via PCR amplification of the regions flanking the target pre-miRNA and their reinsertion into pArgP.

pArg-∆292 was constructed by amplifying the region from pre-miR-290 to pre-miR-291a with primers

F: 5’-CGTAGAGCTCTCTCCACGTTCTTTCCTCCT-3’  (SacI)

R: 5’-CGTATTAATTAAGGAGGTCAAGCTTAAGAACAATC-3’  (PacI)

and inserting the resulting fragment into the SacI-NotI sites of pArgoP. The SacI-PacI sites of the resulting intermediate construct were used to insert the region from from pre-miR-291b to pre-miR-295 PCR amplified with primers

F: 5’-CGTAGAGCTCTTAATTAATGAGAACTCAAAACGGCTAAGA-3’  (SacI - PacI)

R: 5’-CGTAGCGGCCGCCTGCTCCCCAACCCACTAC-3’  (NotI)].

pArg-∆293 was constructed by amplifying the region from pre-miR-290 to pre-miR-291b with primers

F: 5’-CGTAGAGCTCTCTCCACGTTCTTTCCTCCT-3’  (SacI)

R: 5’-CGTACATATGACAGGGACCTGCTGTGGTAAT-3’  (NdeI)

and amplifying the region from pre-miR-294 to pre-miR-295 with primers

F: 5’-CGTACATATGTGCAGTTGGCCTAAGTGTTG-3’  (NdeI)

R: 5’-CGTAGCGGCCGCCTGCTCCCCAACCCACTAC-3’  (NotI)

The resulting fragments were joined via their NdeI sites and inserted into the SacI-NotI sites of pArgP in a single step ligation.

pArg-∆371 was constructed by inserting the PCR amplified pre-miR-372 to pre-miR-373 fragment of pArgF101-miR-371-373 with primers

F: 5’-CGTAGGATCCAACCTGCGGAGAAGATACCA-3’  (SacI)

R: 5’-CGTAGCGGCCGCGGAACCCAATAGACCAGAAACA-3’  (NotI)

into the SacI-NotI sites of pArgP.

**3. miRNA reporters**

**3.1. Reporters with perfectly complementary target sites**

The perfectly complementary target sites were generated by annealing the oligos listed below and inserting the resulting duplexes into the NotI site of pArg. Screening for insertions in the correct orientation was by PCR amplification with primer

5’-TCTAGAGCCTCTGCTAACCA-3’

and the strand of the duplex that had a sense orientation to the miRNA.

**Oligos in the sense orientation with respect to the miRNAs:**

5’-GGCCGGTACTCAAACTATGGGGGCACTTTTTTTT-3’ miR290-5pr-S

5’-GGCCGGTACTCAAACTGTGTGACATTTTGTT-3’ miR293-5pr-S

5’-GGCCAAAAAAAAGTGCCCCCATAGTTTGAGTACC-3’ miR290-5pr-A

5’-GGCCAACAAAATGTCACACAGTTTGAGTACC-3’ miR293-5pr-A

5’-GGCCTAAAAAGTGCCGCCTAGTTTTAAGCCCCGCC-3’ miR290-3pr-S

5’-GGCCAGAAAAGTGCCGCCAGGTTTTGAGTGTCACC-3’ miR292-3pr-S

5’-GGCCTAAGAAGTGCCGCAGAGTTTGTAGTGTTGCC-3’ miR293-3pr-S

5’-GGCCGGCGGGGCTTAAAACTAGGCGGCACTTTTTA-3’ miR290-3pr-A

5’-GGCCGGTGACACTCAAAACCTGGCGGCACTTTTCT-3’ miR292-3pr-A

5’-GGCCGGCAACACTACAAACTCTGCGGCACTTCTTA-3’ miR293-3pr-A

**Oligos antisense to the miRNAs:**

5’-GGCCGAGAAAGTGCTTCCACTTTGTGTGCCACT-3’ miR291a-3pr-S

5’-GGCCGGGAAAGTGCATCCATTTTGTTTGTCTCT-3’ miR291b-3pr-S

5’-GGCCTAGAAAGTGCTACTACTTTTGAGTCTCTCC-3’ miR295-3pr-S

5’-GGCCAGTGGCACACAAAGTGGAAGCACTTTCTC-3’ miR291a-3pr-A

5’-GGCCAGAGACAAACAAAATGGATGCACTTTCCC-3’ miR291b-3pr-A

5’-GGCCGGAGAGACTCAAAAGTAGTAGCACTTTCTA-3’ miR295-3pr-A

5’-GGCCGGCCATCAAAGTGGAGGCCCTCTCTTGA-3’ miR291a-5pr-S

5’-GGCCGTCGATCAAAGTGGAGGCCCTCTCCGCG-3’ miR291b-5pr-S

5’-GGCCGAGACTCAAATGTGGGGCACACTTCTGG-3’ miR295-5pr-S

5’-GGCCTCAAGAGAGGGCCTCCACTTTGATGGCC-3’ miR291a-5pr-A

5’-GGCCCGCGGAGAGGGCCTCCACTTTGATCGAC-3’ miR291b-5pr-A

5’-GGCCCCAGAAGTGTGCCCCACATTTGAGTCTC-3’ miR295-5pr-A

**3.2. Reporters with seed only and bulge target sites**

Seed only and bulge target sites were generated by concatamerization of the oligos listed below (sequences listed as 5’->3’). After 5’-phosphorylation the oligonucleotides were annealed to form End-S/Body-A, End-A/Body-S and Body-A/Body-S duplexes and the resulting double stranded fragments were co-ligated into the BamHI/NotI sites of pArgoP. Constructs containing 4 repeats of the target sites were identified by PCR amplification with primers

F: 5’-TCTAGAGCCTCTGCTAACCA-3’

R: 5’-AGGGAAAAAGATCTCAGTGG-3’

and verified by sequencing.

**Position 8 mutations:**

2-7C-S-End-S:

GATCCAGGCAGACAGAGAGACAG**G**GCACTggTA

2-7C-S-End-A:

GGCCTAccAGTGC**C**CTGTCTCTCTGTCTGCCTG

2-7C-S-Body-S:

CCAGCAGGCAGACAGAGAGACAG**G**GCACTggTA

2-7C-S-Body-A:

CTGGTAccAGTGC**C**CTGTCTCTCTGTCTGCCTG

2-7U-S-End-S:

GATCCAGGCAGACAGAGAGACAGAGGCACTgTA

2-7U-S-End-A:

GGCCTAcAGTGCCTCTGTCTCTCTGTCTGCCTG

2-7U-S-Body-S:

CCAGCAGGCAGACAGAGAGACAGAGGCACTgTA

2-7U-S-Body-A:

CTGGTAcAGTGCCTCTGTCTCTCTGTCTGCCTG

2-7G-S-End-S:

GATCCAGGCAGACAGAGAGACAGGCGGCACgTA

2-7G-S-End-A:

GGCCTAcGTGCCGCCTGTCTCTCTGTCTGCCTG

2-7G-S-Body-S:

CCAGCAGGCAGACAGAGAGACAGGCGGCACgTA

2-7G-S-Body-A:

CTGGTAcGTGCCGCCTGTCTCTCTGTCTGCCTG

2-7A-S-End-S:

GATCCAGGCAGACAGAGAGACAG**T**GCACTTgTA

2-7A-S-End-A:

GGCCTAcAAGTGC**A**CTGTCTCTCTGTCTGCCTG

2-7A-S-Body-S:

CCAGCAGGCAGACAGAGAGACAG**T**GCACTTgTA

2-7A-S-Body-A:

CTGGTAcAAGTGC**A**CTGTCTCTCTGTCTGCCTG

**Position 9 mutations:**

2-7UU-S-End-S:

GATCCAGGCAGACAGAGAGACA**A**AGCACTTgTA

2-7UU-S-End-A:

GGCCTAcAAGTGCT**T**TGTCTCTCTGTCTGCCTG

2-7UU-S-Body-S:

CCAGCAGGCAGACAGAGAGACA**A**AGCACTTgGA

2-7UU-S-Body-A:

CTGGTAcAAGTGCT**T**TGTCTCTCTGTCTGCCTG

3-7UU-S-End-S:

GATCCAGGCAGACAGAGAGACA**A**AGCACTggTA

3-7UU-S-End-A:

GGCCTAccAGTGCT**T**TGTCTCTCTGTCTGCCTG

3-7UU-S-Body-S:

CCAGCAGGCAGACAGAGAGACA**A**AGCACTggTA

3-7UU-S-Body-A:

CTGGTAccAGTGCT**T**TGTCTCTCTGTCTGCCTG

2-7UA-S-End-S:

GATCCAGGCAGACAGAGAGACA**T**AGCACTTgTA

2-7UA-S-End-A:

GGCCTAcAAGTGCT**A**TGTCTCTCTGTCTGCCTG

2-7UA-S-Body-S:

CCAGCAGGCAGACAGAGAGACA**T**AGCACTTgTA

2-7UA-S-Body-A:

CTGGTAcAAGTGCT**A**TGTCTCTCTGTCTGCCTG

3-7UA-S-End-S:

GATCCAGGCAGACAGAGAGACA**T**AGCACTggTA

3-7UA-S-End-A:

GGCCTAccAGTGCT**A**TGTCTCTCTGTCTGCCTG

3-7UA-S-Body-S:

CCAGCAGGCAGACAGAGAGACA**T**AGCACTggTA

3-7UA-S-Body-A:

CTGGTAccAGTGCT**A**TGTCTCTCTGTCTGCCTG

2-7UG-S-End-S:

GATCCAGGCAGACAGAGAGACA**C**AGCACTTgTA

2-7UG-S-End-A:

GGCCTAcAAGTGCT**G**TGTCTCTCTGTCTGCCTG

2-7UG-S-Body-S:

CCAGCAGGCAGACAGAGAGACA**C**AGCACTTgTA

2-7UG-S-Body-A:

CTGGTAcAAGTGCT**G**TGTCTCTCTGTCTGCCTG

3-7UG-S-End-S:

GATCCAGGCAGACAGAGAGACA**C**AGCACTggTA

3-7UG-S-End-A:

GGCCTAccAGTGCT**G**TGTCTCTCTGTCTGCCTG

3-7UG-S-Body-S:

CCAGCAGGCAGACAGAGAGACA**C**AGCACTggTA

3-7UG-S-Body-A:

CTGGTAccAGTGCT**G**TGTCTCTCTGTCTGCCTG

2-7UC-S-End-S:

GATCCAGGCAGACAGAGAGACAGCGGCACTgTA

2-7UC-S-End-A:

GGCCTAcAGTGCCGCTGTCTCTCTGTCTGCCTG

2-7UC-S-Body-S:

CCAGCAGGCAGACAGAGAGACAGCGGCACTgTA

2-7UC-S-Body-A:

CTGGTAcAGTGCCGCTGTCTCTCTGTCTGCCTG

*Note: 2-7UC oligos have the same sequence as 2-7U oligos because the first nucleotide of the randomized sequence is a C.

3-7UC-S-End-S:

GATCCAGGCAGACAGAGAGACA**G**AGCACTggTA

3-7UC-S-End-A:

GGCCTAccAGTGCT**C**TGTCTCTCTGTCTGCCTG

3-7UC-S-Body-S:

CCAGCAGGCAGACAGAGAGACA**G**AGCACTggTA

3-7UC-S-Body-A:

CTGGTAccAGTGCT**C**TGTCTCTCTGTCTGCCTG

**miR-292-3p0, miR-292-3p+1 and miR-293-3p+2 seed only target sites.**

miR-292-3p0 WT target site = 2-7C target site. See sequences above.

3-7C-S-End-S:

GATCCAGGCAGACAGAGAGACAG**G**GCACTggTA

3-7C-S-End-A:

GGCCTAccAGTGC**C**CTGTCTCTCTGTCTGCCTG

3-7C-S-Body-S:

CCAGCAGGCAGACAGAGAGACAG**G**GCACTggTA

3-7C-S-Body-A:

CTGGTAccAGTGC**C**CTGTCTCTCTGTCTGCCTG

3-7CG-S-End-S:

GATCCAGGCAGACAGAGAGACAGCGGCACTgTA

3-7CG-S-End-A:

GGCCTAcAGTGCCGCTGTCTCTCTGTCTGCCTG

3-7CG-S-Body-S:

CCAGCAGGCAGACAGAGAGACAGCGGCACTgTA

3-7CG-S-Body-A:

CTGGTAcAGTGCCGCTGTCTCTCTGTCTGCCTG

4-7CG-S-End-S:

GATCCAGGCAGACAGAGAGACAGCGGCACggTA

4-7CG-S-End-A:

GGCCTAccGTGCCGCTGTCTCTCTGTCTGCCTG

4-7CG-S-Body-S:

CCAGCAGGCAGACAGAGAGACAGCGGCACggTA

4-7CG-S-Body-A:

CTGGTAccGTGCCGCTGTCTCTCTGTCTGCCTG

4-7CGC-S-End-S:

GATCCAGGCAGACAGAGAGACAGGCGGCACgTA

4-7CGC-S-End-A:

GGCCTAcGTGCCGCCTGTCTCTCTGTCTGCCTG

4-7CGC-S-Body-S:

CCAGCAGGCAGACAGAGAGACAGGCGGCACgTA

4-7CGC-S-Body-A:

CTGGTAcGTGCCGCCTGTCTCTCTGTCTGCCTG

5-7CGC-S-End-S:

GATCCAGGCAGACAGAGAGACAGGCGGCAggTA

5-7CGC-S-End-A:

GGCCTAccTGCCGCCTGTCTCTCTGTCTGCCTG

5-7CGC-S-Body-S:

CCAGCAGGCAGACAGAGAGACAGGCGGCAggTA

5-7CGC-S-Body-A:

CTGGTAccTGCCGCCTGTCTCTCTGTCTGCCTG

Key:

**Red:** Cohesive ends. May or may not include restriction sites.

Blue: Randomized sequence/spacer.

Black, uppercase: Regions that pair to miRNA seeds.

Black, lowercase: Regions that mismatch the miRNA seeds.

**BULGE TARGET SITES**

3-7CG-B-End-S:

GATCCACACTCAAAAaca**CGGCACT**gTA

3-7CG-B-End-A:

GGCCTAcAGTGCCGTGTTTTTGAGTGTG

3-7CG-B-Body-S:

CCAGCACACTCAAAAaca**CGGCACT**gTA

3-7CG-B-Body-A:

CTGGTAcAGTGCCGTGTTTTTGAGTGTG

4-7CG-B-End-S:

GATCCACACTCAAAAaca**CGGCAC**ggTA

4-7CG-B-End-A:

GGCCTAccGTGCCGTGTTTTTGAGTGTG

4-7CG-B-Body-S:

CCAGCACACTCAAAAaca**CGGCAC**ggTA

4-7CG-B-Body-A:

CTGGTAccGTGCCGTGTTTTTGAGTGTG

4-7CGC-B-End-S:

GATCCACACTACAAACaa**GCGGCAC**gTA

4-7CGC-B-End-A:

GGCCTA**cGTGCCGC**ttGTTTGTAGTGTG

4-7CGC-B-Body-S:

CCAGCACACTACAAACaa**GCGGCAC**gTA

4-7CGC-B-Body-A:

CTGGTAcGTGCCGCttGTTTGTAGTGTG

5-7CGC-B-End-S:

GATCCACACTACAAACaa**GCGGCA**ggTA

5-7CGC-B-End-A:

GGCCTA**ccTGCCGC**ttGTTTGTAGTGTG

5-7CGC-B-Body-S:

CCAGCACACTACAAACaa**GCGGCA**ggTA

5-7CGC-B-Body-A:

CTGGTAccTGCCGCttGTTTGTAGTGTG

**Key:**

**Red:** Cohesive ends. May or may not include restriction sites.

Blue: Randomized sequence/spacer.

Black, uppercase: Regions that pair to miRNA seeds.

Black, lowercase: Regions that mismatch the miRNA seeds.

Green: Regions that pair to miRNA non-seed regions.

Brown: Bulge sites.
